# SUMO1 modification of KHSRP regulates tumorigenesis by preventing the *TL-G-Rich* miRNA biogenesis

**DOI:** 10.1186/s12943-017-0724-6

**Published:** 2017-10-11

**Authors:** Haihua Yuan, Rong Deng, Xian Zhao, Ran Chen, Guofang Hou, Hailong Zhang, Yanli Wang, Ming Xu, Bin Jiang, Jianxiu Yu

**Affiliations:** 1 Department of Oncology, Shanghai 9th People’s Hospital, Shanghai Jiao Tong University School of Medicine, 280 Mohe Road, Shanghai, 201999 China; 2 Department of Biochemistry and Molecular Cell Biology, Shanghai Key Laboratory of Tumor Microenvironment and Inflammation, Shanghai Jiao Tong University School of Medicine, Shanghai, 200025 China; 3 State Key Laboratory of Oncogenes and Related Genes, Shanghai Jiao Tong University School of Medicine, Shanghai, 200025 China; 4 Department of Pathophysiology, Key Laboratory of Cell Differentiation and Apoptosis of Chinese Ministry of Education, Shanghai JiaoTong University School of Medicine, Shanghai, 200025 China

**Keywords:** KHSRP, SUMO1 modification, *TL-G-Rich* miRNA biogenesis, Tumorgenesis

## Abstract

**Background:**

MicroRNAs (miRNAs) are important regulators involved in diverse physiological and pathological processes including cancer. SUMO (small ubiquitin-like modifier) is a reversible protein modifier. We recently found that SUMOylation of TARBP2 and DGCR8 is involved in the regulation of the miRNA pathway. KHSRP is a single stranded nucleic acid binding protein with roles in transcription and mRNA decay, and it is also a component of the Drosha-DGCR8 complex promoting the miRNA biogenesis.

**Methods:**

The in vivo SUMOylation assay using the Ni^2+^-NTA affinity pulldown or immunoprecipitation (IP) and the in vitro *E.coli*-based SUMOylation assay were used to analyze SUMOylation of KHSRP. Nuclear/Cytosol fractionation assay and immunofluorescent staining were used to observe the localization of KHSRP. High-throughput miRNA sequencing, quantantive RT-PCR and RNA immunoprecipitation assay (RIP) were employed to determine the effects of KHSRP SUMO1 modification on the miRNA biogenesis. The soft-agar colony formation, migration, vasculogenic mimicry (VM) and three-dimensional (3D) cell culture assays were performed to detect the phenotypes of tumor cells in vitro, and the xenograft tumor model in mice was conducted to verify that SUMO1 modification of KHSRP regulated tumorigenesis in vivo.

**Results:**

KHSRP is modified by SUMO1 at the major site K87, and this modification can be increased upon the microenvironmental hypoxia while reduced by the treatment with growth factors. SUMO1 modification of KHSRP inhibits its interaction with the pri-miRNA/Drosha-DGCR8 complex and probably increases its translocation from the nucleus to the cytoplasm. Consequently, SUMO1 modification of KHSRP impairs the processing step of pre-miRNAs from pri-miRNAs which especially harbor short G-rich stretches in their terminal loops (TL), resulting in the downregulation of a subset of *TL-G-Rich* miRNAs such as let-7 family and consequential tumorigenesis.

**Conclusions:**

Our data demonstrate how the miRNA biogenesis pathway is connected to tumorigenesis and cancer progression through the reversible SUMO1 modification of KHSRP.

**Electronic supplementary material:**

The online version of this article (10.1186/s12943-017-0724-6) contains supplementary material, which is available to authorized users.

## Background

MicroRNAs (miRNAs) have very important roles in the regulation of gene expression. The miRNA biogenesis is a multistep processes in mammalian. Conventionally, miRNAs are firstly transcribed by RNA polymerases II and III as primary miRNAs (pri-miRNAs) [1]. These pri-miRNAs are later processed to the 65-nucleotide (nt) hairpin precursor miRNAs (pre-miRNAs) by the microprocessor complex (MC), which is mainly composed of Drosha and DGCR8 [2]. Then pre-miRNAs are transported by Exportin-5/Ran-GTP from the nucleus to the cytoplasm [3], where further they are accurately cleaved into an ~20–25 bp double-stranded mature miRNAs by the Dicer-TARBP2 complex [4]. The mature miRNA, one strand of the duplex, and Argonaute (Ago) proteins mainly constitute a RNA-induced silencing complex (RISC), which mediates posttranscriptional gene silencing [5, 6].

KHSRP, a hnRNP K homology (KH)-type splicing regulatory protein, has been identified as a main component of the Drosha complex to promote the biogenesis of a select group of miRNAs [7, 8]. KHSRP contains four KH domains that bind to the single-stranded nucleic acids, and specifically to short G-rich stretches in the terminal loop (*TL-G-rich*) of primary/precursor miRNAs, which favors the maturation of a subset of miRNAs including let-7 family [7–10]. In addition, KHSRP also participates in pre-mRNA splicing [11, 12], mRNA decay [13, 14]. KHSRP seems to contain a nuclear localization signal (NLS) which can mediate its shuttling between the nucleus and the cytoplasm [7, 15, 16], however the mechanism underlying the translocation of KHSRP is unclear.

SUMO (small ubiquitin-like modifier) is a reversible protein modifier, including SUMO 1–4 in human [17, 18]. SUMOylation plays critical roles in a variety of cellular processes through regulating the activity [19], stability [20] or localization [21] of target proteins. In particular, SUMOylation can promote the target protein nuclear import, for example, SUMOylated RanGAP1 mostly localizes in the nucleus while the unmodified RanGAP1 is cytosolic [22, 23]. However SUMOylation can also increase the target protein nuclear export, for instance, SUMOylation of p53 promotes its nuclear export [24, 25]. Recently we have reported that SUMOylation of TARBP2 and DGCR8 is involved in the regulation of the miRNA pathway [20, 26, 27].

Here we identified that KHSRP was modified by SUMO1 at the major site K87 in vitro and in cells for the first time. We found that KHSRP SUMOylation was upregulated by the microenvironmental hypoxia while downregulated by growth factors. SUMOylation could facilitate KHSRP translocation from the nucleus to the cytoplasm. More importantly, KHSRP SUMOylation inhibited the biogenesis of a subset of *TL-G-rich* miRNAs, which was probably contributed to SUMOylation promoting the disassociation of KHSRP from the pri-miRNA/Drosha-DGCR8 complex. Furthermore, we observed that the dysregulation of *TL-G-Rich* miRNAs such as the members of let-7 family mediated by KHSRP SUMOylation was linked to tumorigenesis and cancer progression.

## Results

### KHSRP is SUMOylated in cells and in vitro

**Fig. 1:**
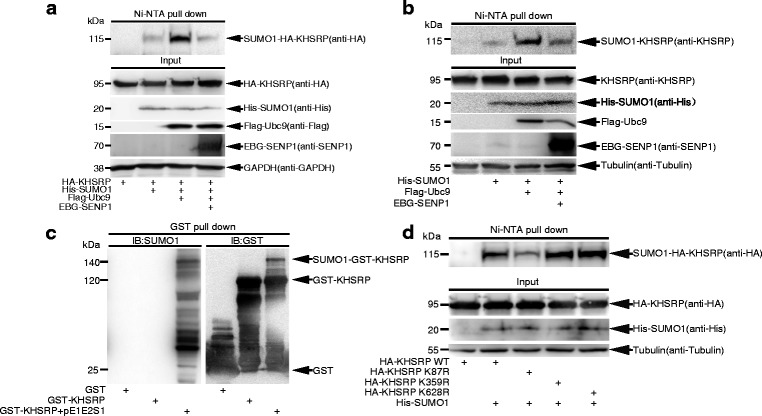
KHSRP is modified by SUMO1 at the major site K87 in vitro and in cells. **a-b** Exogenous and endogenous KHSRP in cells are modified by SUMO1. 293T cells transfected with indicated plasmids were lysed and pulled down with Ni^2+^-NTA resin for SUMOylation assay, and SUMO1 modification of KHSRP was analyzed by Western blotting with indicated antibodies. **c** SUMO1 modification of KHSRP is verified by in vitro *E.coli*-based SUMOylation assay. Plasmid pGEX-4T-1-KHSRP was co-transformed with or without pE1E2SUMO1 plasmid into *E.coli* BL21 (DE3). After GST pull-down purification, Western blotting was conducted with anti-SUMO1 antibody and the same membrane was detected with anti-GST antibody after stripping. **d** Mutation of K87R weakens SUMO1 modification of KHSRP in 293T cells. The construct pEF-5HA-KHSRP-WT, or -K87R, or -K359R, or -K628R was co-transfected with His-SUMO1 into 293T cells. 48 h after transfection, cells were lysed for the SUMOylation assay with Ni^2+^-NTA resin

To detect whether KHSRP can be SUMOylated in cells, we co-transfected His-SUMO1, Flag-Ubc9 and EBG-SENP1 with (Fig. 1a) or without (Fig. 1b) HA-KHSRP plasmid into 293T cells, and performed the SUMOylation assay by using the method of Ni^2+^-NTA resin pull-down [28]. The results showed that both exogenous and endogenous KHSRP were SUMOylated even with only His-SUMO1. The SUMO1 modification of KHSRP was strongly enhanced by the E2 conjugating enzyme Ubc9, whereas it was attenuated with the addition of the de-SUMOylation enzyme SENP1. Next, we preformed an in vitro *E.coli*-based SUMOylation assay [29] and showed that GST-KHSRP was SUMOylated in the *E.coli* transformed with pE1E2SUMO1 and GST-KHSRP but not in the *E.coli* transformed with GST or GST-KHSRP alone (Fig. 1c). More importantly, we further proved that SUMO1 modification of KHSRP occurred naturally in 293T cells by the method of immunoprecipitation (IP). Endogenous SUMO1-KHSRP was detected only in the immunoprecipitated complexes with anti-KHSRP antibody but not with normal IgG (Additional file 1: Fig. S1). Collectively, these results demonstrate that KHSRP can be modified by SUMO1 in cells and in vitro.

### K87 is a major SUMO-site of KHSRP

According to the prediction of SUMOplot™ Analysis Program, KHSRP has several putative SUMOylated sites (Additional file 2: Fig. S2). To determine which lysines (Ks) of KHSRP are the major sites for SUMOylation, we mutated those sites with R (arginine) replacing K, respectively. Wild type (WT) or mutant HA-KHSRP construct with His-SUMO1 were co-transfected into 293T cells for the Ni^2+^-NTA precipitation SUMOylation assays. The results showed that the mutation K87R clearly reduced the SUMOylation level of KHSRP compared with HA-KHSRP-WT and other point mutations including -K359R, −K628R (Fig. 1d), −K244R, −K251R, −K435R, −K473R and -K494R (Additional file 3: Fig. S3). However SUMO1 modification was not removed completely in KHSRP K87R mutant, we speculated there might exist other sites apart from the eight putative sites predicted by SUMOplot™ Analysis Program. Taken together, these results suggest that K87 is a major SUMO1 modification site of KHSRP.

### SUMOylation of KHSRP is regulated by hypoxia, hydrogen peroxide and growth factors

**Fig. 2:**
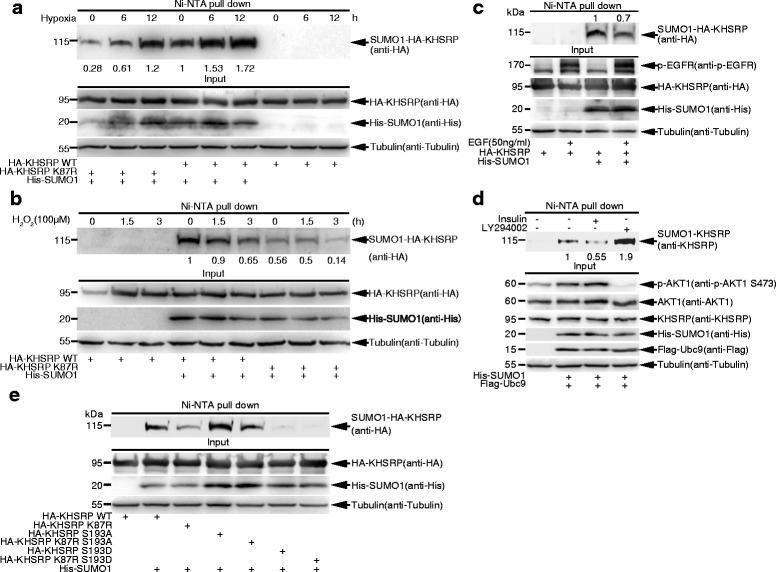
SUMO1 modification of KHSRP is regulated by hypoxia, hydrogen peroxide and growth factors. **a** Hypoxia upregulates SUMO1 modification of KHSRP. 293T cells transfected with His-SUMO1 and HA-KHSRP-WT or HA-KHSRP-K87R were cultured in 1% oxygen condition (hypoxia) for 6, 12 h before cells were harvested. Ni^2+^-NTA resin pull down was performed to detect the SUMO1 modification of exogenous KHSRP. **b** Hydrogen peroxide (H_2_O_2_) downregulates SUMO1 modification of KHSRP. 293T cells transfected with His-SUMO1 and HA-KHSRP-WT or HA-KHSRP-K87R were treated with 100 μM of H_2_O_2_ for 1.5, 3 h before cells were harvested. Ni^2+^-NTA resin pull down was performed to detect the SUMO1 modification of KHSRP. **c** EGF downregulates SUMO1 modification of KHSRP. 36 h after transfection with His-SUMO1 and HA-KHSRP-WT, 293T cells were starved overnight and then stimulated with EGF (50 ng/ml) for 5 min before lysed for Ni^2+^-NTA pull down, and followed by Western blotting with indicated antibodies. **d** Insulin downregulates SUMO1 modification of KHSRP. 293T cells transfected with His-SUMO1 and Flag-Ubc9 were treated with insulin (1 μM) for 1 h or LY294002 (25 μM) for 16 h before cells were harvested. Ni^2+^-NTA resin pull down was performed to detect the SUMO1 modification of endogenous KHSRP. p-AKT1 (S473) antibody was used to detect the phosphorylation level of AKT1. **e** Phosphorylation of KHSRP downregulates SUMO1 modifcation of KHSRP. 293T cells transfected with His-SUMO1 and HA-KHSRP-WT, −K87R, −S193A, −K87R-S193A, −S193D, or -K87R-S193D were lysed for the SUMOylation assay with the Ni^2+^-NTA resin pull down. The band intensities were calculated by ImageJ software and the ratios were quantified (**a-d**)

Since hypoxia can regulate the SUMOylation level of distinct SUMO targets [26, 30], we investigated whether oxidative stress can regulate the SUMOylation level of KHSRP. Indeed, we found that the SUMO1 modification level of exogenous KHSRP was significantly enhanced while those of KHSRP-K87R was less increased by hypoxia (1% O_2_) treatment for 6 and 12 h (Fig. 2a). In contrast to hypoxia, the treatment with 100 μM of hydrogen peroxide (H_2_O_2_) strongly decreased the SUMO1 modification level of KHSRP at 1.5 and 3 h whereas slightly affected that of KHSRP-K87R (Fig. 2b).

We also found that EGF slightly downregulated SUMO1 modification of KHSRP (Fig. 2c, lane 4), which is consistent with a rapid alteration in SUMOylation of target proteins under EGF stimulation [31, 32]. Since activated AKT by growth signals can phosphorylate KHSRP at serine 193 (S193) [33], we wanted to test whether this phosphorylation influences SUMO1 modification of KHSRP. Interestingly, the SUMO1 modification level of KHSRP was attenuated when AKT1 phosphorylation was induced by the treatment with insulin (Fig. 2d, lane 3). On the contrary, SUMO1 modification of KHSRP was significantly increased by inhibition of AKT1 phosphorylation via the treatment with LY294002 (Fig. 2d, lane 4). Moreover, the phospho-site mutant KHSRP-S193A significantly increased the SUMO1 modification level of KHSRP (Fig. 2e, lanes 4, 5), whereas the phosphomimetic mutant KHSRP-S193D almost abolished its SUMOylation (Fig. 2E, lanes 6, 7), as expectedly. Thus, the above results suggest that SUMO1 modification of KHSRP can be regulated by external signaling pathways, especially PI3K/AKT1 signal pathway.

### SUMOylation of KHSRP promotes tumorigenesis and cancer progression

**Fig. 3:**
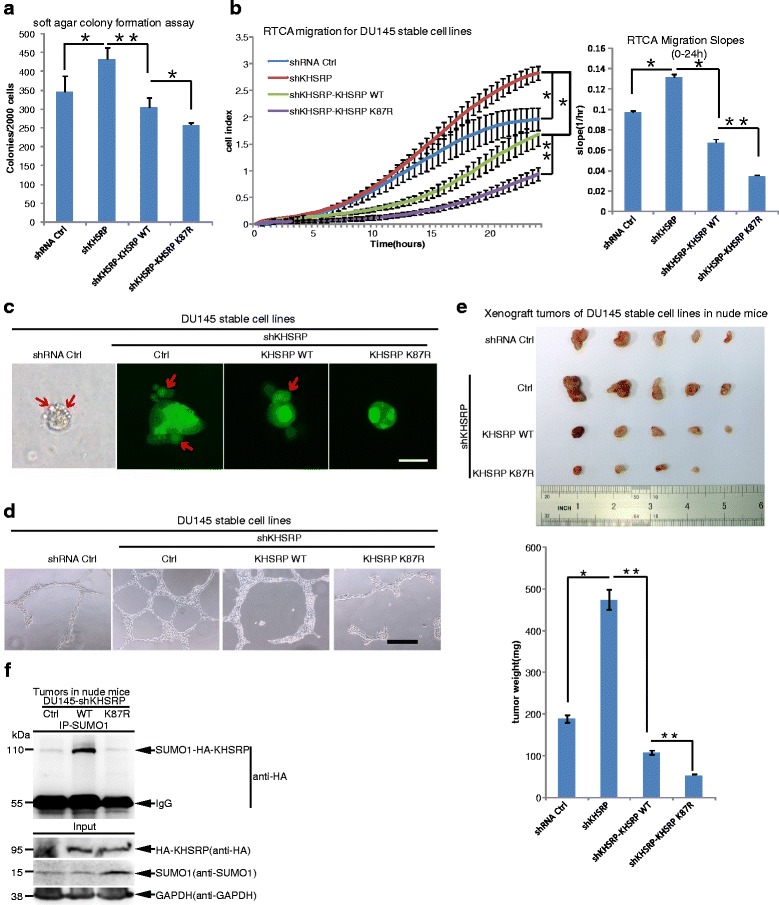
SUMOylation of KHSRP is involved in tumorigenesis. **a** KHSRP-K87R downregulates the anchorage-independent growth in DU145 stable cell lines. In soft agar colony forming assays, stable cell lines DU145 shRNA control, shKHSRP, shKHSRP-KHSRP-WT or shKHSRP-KHSRP-K87R were seeded in 2 ml of medium containing 5% FBS with 0.35% agar at 2 × 10^3^ cells/well and layered onto the base. The photographs were taken 21 days later and the number of colonies was scored. **b** KHSRP-K87R downregulates the migration ability in DU145 stable cell lines. The RTCA migration assay was performed to detect the migration ability in above stable DU145 cell lines with xCELLigene RTCA-DP instrument. The kinetic cell index of their migration was recorded every 15 min for 24 h (left panel) and the relative slope value was calculated (right panel). **c** KHSRP-K87R downregulates the invasive ability in above stable DU145 cell lines. The 3D–culture assay was performed to detect the invasive ability of DU145 stable cell lines. The photos were taken at day 7. The first image was taken under the white light, and the green signals indicates the expression of GFP (Green Fluorescent Protein) in the plasmid CD513B-HA-KHSRP. **d** KHSRP-K87R downregulates the aggressive ability in DU145 stable cell lines in vasculogenic mimicry (VM) assay. VM assay was performed to detect the aggressive ability in above stable DU145 cell lines. The photos were taken 20 h later. Scale: 500 μm. Independent experiments (**a**-**d**) were repeated three times. **e** KHSRP-K87R suppresses xenograft tumor growth in vivo. 5 male BALB/c nude mice were injected subcutaneously with stable DU145 cell lines (2.5 × 10^6^ cells/each) expressing the shRNA control in the left back and shKHSRP in the right back, respectively. Another 5 male BALB/c nude mice were injected subcutaneously with stable DU145 cell line expressing shKHSRP-KHSRP-WT in the left back and shKHSRP-KHSRP-K87R in the right back, respectively. Mice were sacrificed 5 weeks later, and tumors were dissected (upper panel) and assessed by weight (low panel). (**a**-**d**) **f** KHSRP SUMOylation could be detected in tumors of nude mice. The tumors of nude mice, which was chosen from the groups of shKHSRP, shKHSRP-KHSRP-WT or -K87R, were lysed in NEM-RIPA as described in the Methods. The proteins was immunoprecipitated by anti-SUMO1 antibody, Western blotting was detected with anti-HA antibody

Hypoxia is linked to poor patient outcomes and has a negative impact on the effectiveness of tumor treatment, such as surgery and radiotherapy [34]. The signaling pathway induced by EGF or insulin are also associated with tumor cell proliferation and migration [35, 36]. KHSRP knockdown in glioblastoma multiforme (GBM) cells promotes migration and causes multifocal tumor in a mouse model. 70% of GBM patients (*n* = 548) with high expression levels of KHSRP survive long after surgery [37]. KHSRP knockdown in osteosarcoma cell U2OS significantly upregulates cell proliferation [7]. All these suggest that KHSRP behaves as a tumor suppressor. Since our above data have proven that KHSRP SUMOylation could be regulated by hypoxia and growth factors, we questioned whether K87-SUMOylation of KHSRP is involved in tumorigenesis and cancer progression. To this end, we constructed stable cell lines, in which endogenous KHSRP was firstly stably knockdown by a short hairpin RNA targeting KHSRP 3’UTR (shKHSRP) in the lentiviral vector pLKO.1 system, and then HA-KHSRP-WT and HA-KHSRP-K87R were re-introduced by the lentiviral-expressing system. The expression levels of endogenous KHSRP and re-expressed HA-KHSRP-WT or HA-KHSRP-K87R were assessed by Western blotting (Additional file 4: Fig. S4). To explore whether SUMOylation of KHSRP affects the transforming potential of each stable DU145 cell lines, we performed a soft-agar colony-forming assay. We found that knockdown of KHSRP enhanced the capacity of anchorage-independent growth, whereas re-expression of the HA-KHSRP-WT suppressed soft-agar colony formation in DU145 cells as expectedly (Fig. 3a). But interestingly, re-expression of the SUMO-site mutant HA-KHSRP-K87R more significantly inhibited the anchorage-independent growth compared with the HA-KHSRP-WT (Fig. 3a). These results indicate that SUMOylation inhibits the tumor-suppressive role of KHSRP.

In order to assess whether SUMOylation of KHSRP influences the migration capacity of tumor cells, we performed a RTCA-migration assay [38]. The results showed that DU145-shKHSRP cells displayed a sharper curve in cell index and a higher slope value of migration compared to that of DU145-shRNA-Ctrl cells, which indicated that KHSRP suppressed tumor cell migration. Re-expression of KHSRP-WT or KHSRP-K87R mutant in DU145-shKHSRP cells both decreased the migratory capability and the latter more powerfully (Fig. 3b). Furthermore, we used the method of 3D growth cell cultures on extracellular matrix to mimic the in vivo conditions to investigate the invasive ability of tumor cells [39]. DU145-shKHSRP cells grew diffusely and displayed a scattered morphology, showing the better capability to invade into extracellular matrix than that of DU145-shRNA control cells, which revealed that KHSRP knockdown increased the invasive ability. On the contrary, ectopic re-expression of KHSRP-WT or KHSRP-K87R in DU145-shKHSRP cells decreased cell penetrating into the matrix; in particular, cells re-expressing KHSRP-K87R grew into tighter and round colonies (Fig. 3c), which suggested the disruption of KHSRP SUMOylation potentially decreased the invasive ability of tumor cells. Aggressive cancer cells forming de novo vascular networks is defined as tumor cell vasculogenic mimicry (VM), which is considered to be an indicator of the aggressive, metastatic phenotype [40]. Therefore, we also assessed whether KHSRP SUMOylation influences VM formation with above four stable DU145 cell lines. The VM assays showed that knockdown of KHSRP in DU145 cells promoted formation of vasculogenic networks on the matrigel compared to that of DU145-shRNA control cells, while re-expression of either KHSRP-WT or KHSRP-K87R greatly attenuated the formation of pipe-like structures on the matrigel and the latter almost abolished the VM formation (Fig. 3d). Moreover, to investigate whether KHSRP SUMOylation affects xenograft tumor growth in vivo, DU145 stable cell lines were inoculated subcutaneously into the backs of nude mice. Tumor growth measurement was performed at day 15, 21, 27, and 32 after injection, showing that tumors of the DU145-shKHSRP group grew most quickly, whereas tumors of the DU145-shKHSRP re-expressing KHSRP-K87R group grew more slowly than other groups (Additional file 5: Fig. S5). Tumors were weighed after killing the nude mice at 5 weeks after injection (Fig. 3e), showing the similar pattern of results as in above soft-agar colony formation, RTCA-migration, 3D culture growth and VM formation. Moreover, SUMO1 modification of KHSRP was confirmed in the xenograft tumors from the KHSRP-WT group but not in the KHSRP-K87R group (Fig. 3f).

To pursue the probability of an correlation between KHSRP SUMOylation and prostate cancer, we further extracted data from the database of “The Cancer Genome Atlas Research Network” (TCGA). Gleason score is a risk stratification factor and prognostic factor, which is positively related to clinical recurrence and metastasis of prostate cancer [41, 42]. The data of KHSRP expression levels together with the associated clinical data revealed that the KHSRP expression levels for 435 prostate cancer patients (Additional file 6: Table S1) was not related to Gleason score (Pearson correlation with r value = 0.056; *P* = 0.242). But among 212 prostate cancer patients with high expression levels of KHSRP (median value >8732 FPKM, see in Table S1), the expression levels of Ubc9, which is only E2 conjugating enzyme for SUMOylation (Fig. 1a-b), was positively correlated with Gleason score (Pearson correlation with r value = 0.147^*^; *P* = 0.033). We also detected the SUMO1 modification of KHSRP in tumors and paracancerous tissues of gastric and colorectal cancer, respectively. The results demonstrated that KHSRP was expressed in tumors higher than that in paracancerous tissues, and SUMO1 modification of KHSRP could be detected only in tumors (Additional file 7: Fig. S6).

Taken together, these data demonstrate that SUMOylation of KHSRP promotes tumorigenesis and cancer progression.

### SUMOylation of KHSRP at K87 inhibits the biogenesis of *TL-G-Rich* miRNAs

**Fig. 4:**
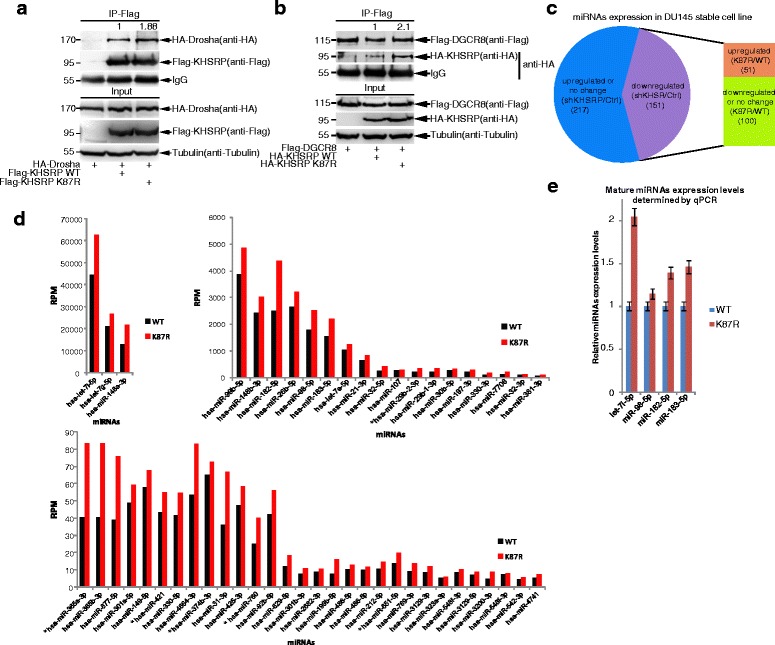
SUMOylation of KHSRP at K87 inhibits the biogenesis of miRNAs. (**A-B)** The mutation K87R of KHSRP increases its interaction with Drosha and DGCR8. 293T cells were transfected with HA-Drosha and Flag-KHSRP-WT or Flag-KHSRP-K87R **(a)**, or transfected with Flag-DGCR8 and HA-KHSRP-WT or HA-KHSRP -K87R **(b)**. 48 h after transfection, cells were lysed for immunoprecipitation with anti-Flag antibody, and followed by Western blotting with indicated antibodies. The band intensities were calculated by ImageJ software and the ratios were quantified. **c** KHSRP SUMOylation regulates the biogenesis of a subset of miRNAs. High-throughput deep sequencing data showed that there were 368 miRNAs whose RPM (reads per million) > 10 RPM in all expressed miRNAs of DU145 control cell line. 151 of these miRNAs whose expression in DU145-shKHSRP cells were downregulated compared to those in DU145-shRNA control cells. 51 of 151 miRNAs whose expression were upregulated in DU145 shKHSRP-KHSRP-K87R cells compared to those in DU145 shKHSRP-KHSRP-WT cells. **d** KHSRP-K87R promotes some miRNAs biogenesis. According to sequencing data, a total of 51 miRNAs whose expression were upregulated in DU145 shKHSRP-KHSRP-K87R cells compared to those in DU145 shKHSRP-KHSRP-WT cells. Among 51 miRNAs, there were only 6 miRNAs which harbor only one single G or none G (labeled with *). Detailed can be seen Additional file 10: Table S4. **e** The biogenesis of some miRNAs promoted by KHSRP-K87R was validated by qRT-PCR. qRT-PCR was performed to assess the expression of endogenous let-7i-5p, miR-98-5p, miR-182a-5p and miR-183a-5p in DU145 shKHSRP-KHSRP-WT and shKHSRP-KHSRP-K87R stable cell lines

Since KHSRP is a component of the Drosha-DGCR8 multiprotein complex [7], we speculated that KHSRP SUMOylation might affect the interaction between KHSRP with the Drosha-DGCR8 complex. 293T cells co-transfected HA-Drosha and Flag-tagged KHSRP-WT or KHSRP-K87R were lysed for immunoprecipitation with anti-Flag antibody and then immunoblotted with anti-HA antibody. The result showed that the Drosha band pulled-down by the SUMO-site mutant KHSRP-K87R was stronger than that by the KHSRP-WT (Fig. 4a). Similarly, we found that the interaction of DGCR8 with the mutant KHSRP-K87R was also stronger than that of DGCR8 with KHSRP-WT (Fig. 4b). Since KHSRP knockdown can abrogate the interaction between Drosha and some special pri-miRNAs, which harbor short G-rich stretches in the terminal loop, thus influencing the biogenesis of those miRNAs [7], and SUMOylation of KHSRP potentially affected its interaction with the Drosha-DGCR8 complex, we raised a question whether KHSRP SUMOylation regulates the biogenesis of *TL-G-Rich* miRNAs. Therefore, next we performed the high-throughput deep sequencing for above DU145 stable cell lines. In DU145-shRNA-Control and DU145-shRNA-KHSRP cells there were 368 miRNAs, in which the expression level of each miRNA >10 reads per million (RPM) (Additional file 8: Table S2). We found that a total of 151 miRNAs were downregulated in DU145 shKHSRP cell lines compared to those in DU145-shRNA-Control cell lines (Fig. 4c; Additional file 9: Table S3). The biogenesis of these miRNAs was considered to be regulated by KHSRP. Consistently, let-7 family and other miRNAs as KHSRP-dependent miRNAs whose precursor terminal loops harbor short G-rich stretches [7, 8], were included in the above set of miRNAs. In particular, of 151 KHSRP-dependent miRNAs, there were 51 miRNAs (including let-7 family let-7i, let-7e, let-7g and miR-98 etc.) that were upregulated in DU145-shRNA-KHSRP cell lines stably re-expressing KHSRP-K87R compared to those of re-expressing KHSRP-WT (Fig. 4c and d; Additional file 10: Table S4), indicating that the mutant KHSRP-K87R promotes the production of a subset of miRNAs. To validate the sequencing results, some miRNAs including let-7i-5p, miR-98-5p, miR-182-5p and miR-183-5p were chosen for validation by using the quantitative RT-PCR. Indeed, the relative expression levels of all these miRNAs were higher in KHSRP-K87R cells compared with cells expressing KHSRP-WT (Fig. 4e). Thus, above results indicate that SUMOylation of KHSRP may reduce its interaction with the pri-miRNA/Drosha-DGCR8 complex, thereby decreasing the biogenesis of *TL-G-Rich* miRNAs.

### SUMOylation of KHSRP promotes its cytoplasmic localization

**Fig. 5:**
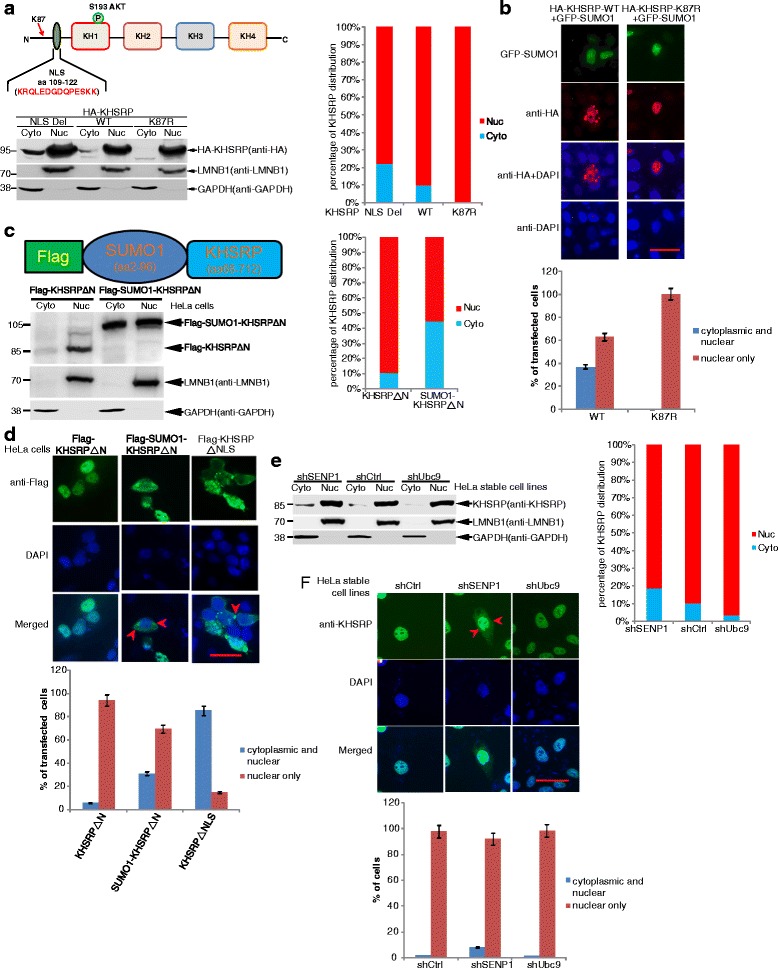
SUMO1 modification of KHSRP promotes its cytoplasmic localization. **a** K87R mutation affects the localization of HA-KHSRP. HeLa cells were transfected with HA-KHSRP-WT, HA-KHSRP-K87R or HA-KHSRP-ΔNLS, respectively. Nuclear and cytosolic fractions were extracted by the Nuclear/Cytosol fractionation kit. Nuclear and cytoplasmic fraction extracts were immunoblotted with indicated antibodies. **b** SUMO1 modification affects the localization of HA-KHSRP by using the method of immunofluorescent staining. HeLa cells transfected with HA-KHSRP-WT or HA-KHSRP-K87R with GFP-SUMO1 were stained with the primary antibody anti-HA (Rabbit), and then with the second antibody of Alexa Fluor 568 anti-rabbit. DAPI staining was to visualize the nucleus. The green signals indicated the expression of GFP-SUMO1 carrying Green Fluorescent Protein and the images of GFP-SUMO1 were directly taken without staining. All the images were taken by Nikon microscope. Scale bar, 25 μm. **c-d** SUMO1 modification of KHSRP promotes its cytosolic localization. Flag-SUMO1-KHSRPΔN was constructed by replacing the N-terminal (aa 1–67) of KHSRP with Flag-tagged SUMO1(aa 2–96). **c** HeLa cells transfected with Flag-KHSRPΔN or Flag-SUMO1-KHSRPΔN were extracted by the Nuclear/Cytosol fractionation kit, and followed by immunoblotting with indicated antibodies. **d** HeLa cells transfected with Flag-KHSRPΔN, Flag-SUMO1-KHSRPΔN or Flag-KHSRPΔNLS were stained using the primary antibody of anti-Flag M2 and the secondary antibody of Alexa Fluor 488 (anti-mouse). **e-f** SUMOylation increases the cytosolic localization of endogenous KHSRP. **e** HeLa-shControl, HeLa-shSENP1 and HeLa-shUbc9 cells were extracted by the Nuclear/Cytosol fractionation kit, then immunoblotted with indicated antibodies. **f** These three cell lines were stained using the primary antibody anti-KHSRP (Rabbit) and the secondary antibody Alexa Fluor 488 (anti-rabbit). Images were taken by Nikon microscope, and the cytoplasmic location of KHSRP was indicated by red arrows. The same scale bar (25 μm) was used in all images (**b, d, f**). The staining cells numbers were counted with the Image J software and the statistical analysis was performed (**b, d, f**). The band intensities were calculated by Image J and quantified by normalization to GAPDH and LMNB1, the percentage of nuclear/cytosolic fraction of every sample was calculated (**a, c, e**)

Since SUMOylation regulates the translocation of many proteins between nucleus and cytoplasm [25, 43, 44], we questioned whether SUMOylation of KHSRP regulates its subcellular localization. The amino acid (aa) sequences from 109 to 122 ‘KRQLEDGDQPESKK’ of KHSRP has been identified as a bipartite NLS (nuclear localization sequence) [11]. However GFP-KH1–4 even with the deletion of NLS still can localize in both the nucleus and the cytoplasm [11, 33], so it is still not very clear about the mechanisms in regulation of the nuclear localization of KHSRP. We performed the nuclear/cytosolic fractionation assays and showed that the deletion of NLS increased the localization of KHSRP in the cytoplasm compared with KHSRP-WT (Fig. 5a, lanes 1, 3), which mostly localized in the nucleus (lanes 2, 4), whereas the SUMO-site mutant KHSRP-K87R almost completely localized in the nucleus and barely in cytoplasm (lanes 5, 6). The cytoplasmic fraction percentage of KHSRPΔNLS, −WT and -K87R was ~22%, ~10% and 0.5%, respectively (Fig. 5a, right panel). The mutant HA-KHSRP-K87R even co-transfected with GFP-SUMO1 still existed only in the nucleus; however in this case of HA-KHSRP-WT co-transfected with GFP-SUMO1, the strong translocation of KHSRP from the nucleus to the cytoplasm in about 39% cells was observed (Fig. 5b and Additional file 11: Fig. S7). These results suggest that SUMO1 modification of KHSRP may control its translocation between nucleus and cytoplasm.

To further confirm SUMOylation of KHSRP affecting its translocation, we generated a mimic SUMOylated KHSRP construct Flag-SUMO1-KHSRPΔN, which the N-terminal (1–67 aa) of KHSRP was replaced by Flag-tagged SUMO1(2–96 aa), to simulate the high SUMO1 modification status of KHSRP. HeLa cells transfected with Flag-KHSRPΔN or Flag-SUMO1-KHSRPΔN (Additional file 12: Fig. S8) were lysed for the nuclear/cytosolic fractionation assay, showing that the cytoplasmic fraction of Flag-SUMO1-KHSRPΔN had higher percentage by ~45% whereas Flag-KHSRPΔN had lower percentage by ~10% (Fig. 5c). Being consistent with this, the distribution in both the cytoplasm and the nucleus of SUMO1-KHSRPΔN was obviously observed in 31% cells whereas that of KHSRPΔN was observed in 5.8% cells. As expectedly, the distribution in both cytoplasma and nucleus of KHSRPΔNLS was in 85% cells (Fig. 5d). Since hypoxia and LY294002 could promote KHSRP SUMOylation, we wanted to detect whether the cytoplamsic/nuclear localization of KHSRP is changed under hypoxia environment and LY294002 stimulation. Consistent with above results, we found that both hypoxia (Additional file 13: Fig. S9) and LY294002 (Additional file 14: Fig. S10) promoted cytoplasmic localization of endogenous KHSRP. These results supported that SUMOylation of KHSRP promotes its cytoplasmic localization by probably facilitating its nuclear export.

Next we attempted to validate the above hypothesis that SUMOylation of KHSRP influences the nuclear localization of KHSRP, we constructed SENP1- (a deSUMOylation enzyme) and Ubc9- (a SUMOylation E2) knockdown HeLa stable cell lines (Additional file 15: Fig. S11) to detect the translocation change of endogenous KHSRP under the status of high-SUMOylation (shSENP1) or low-SUMOylation (shUbc9). The nuclear/cytosol fractionation assays displayed that the fraction of KHSRP in cytoplasm was much higher in HeLa-shSENP1 cells whereas not easily detected in either HeLa-shCtrl or HeLa-shUbc9 cells (Fig. 5e). The cytoplasmic fraction of endogenous KHSRP in HeLa-shSENP1 had higher percentage by ~18.8% whereas that in HeLa-shCtrl and HeLa-shUBC9 had lower percentage by ~10.4% and ~3.8%, respectively (Fig. 5e, right panel). The immunofluorescent staining results also showed that there was a small percentage of KHSRP existed in the cytoplasm in addition to most of KHSRP in the nucleus in HeLa-shSENP1 cells, while KHSRP seemed to be observed almost in the nucleus in HeLa shCtrl or HeLa-shUbc9 cells (Fig. 5f). The distribution in both the cytoplasm and the nucleus of KHSRP was observed in 8% HeLa-shSENP1 cells while that was observed in about 2% HeLa-shRNA Ctrl and shUbc9 cells (Fig. 5f, bottom panel). These data revealed that high SUMOylation of KHSRP promotes its nuclear export to the cytoplasm. Taken together, above results demonstrate that SUMOylation of KHSRP increases its cytoplasmic localization by promoting its nuclear export.

### SUMOylation of KHSRP interferes its interaction with pri-miRNAs

**Fig. 6:**
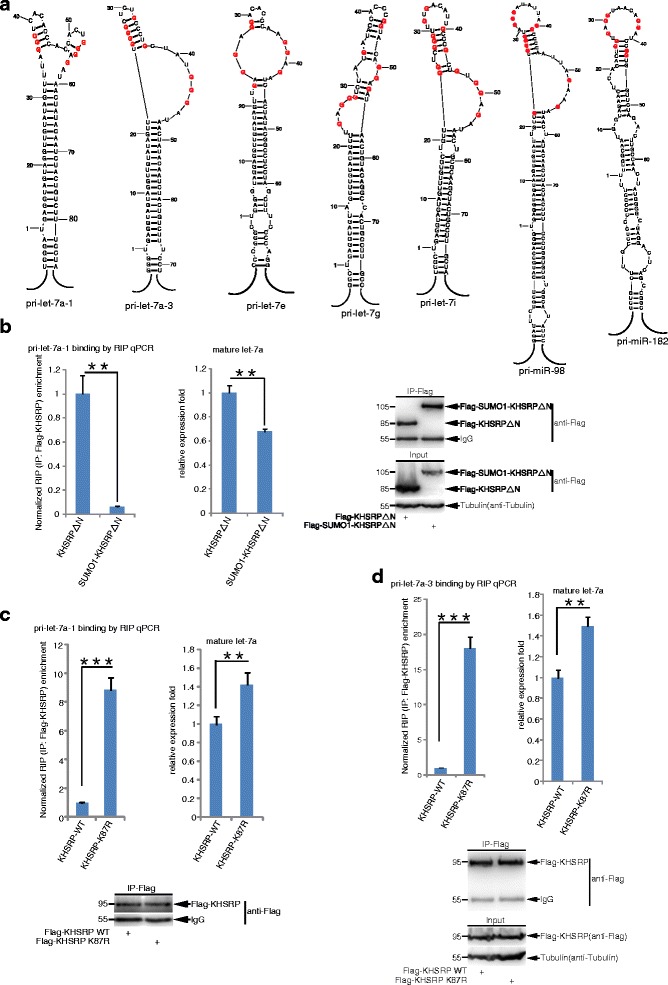
SUMO1 modification of KHSRP interferes its interaction with pri-miRNAs. **a** Pri-miRNAs whose mature miRNAs were upregulated by KHSRP-K87R were analyzed by using the RNAstructure software. All of pri-let-7e, pri-let-7g, pri-let-7i, pri-miR-98 and pri-miR-182 harbored G-rich stretches in their terminal loops, as like pri-let-7a-1 and pri-let-7a-3. **b** KHSRPΔN fusing with SUMO1 decreases its interaction with pri-let-7a-1 and the mature let-7a production. 293T cells transfected CD513B-pri-let-7a-1 and Flag-KHSRPΔN, or Flag-SUMO1-KHSRPΔN were lysed for RIP with anti-Flag antibody, then treated with Trizol, and followed by qRT-PCR for pri-let-7a-1. The relative recruitment fold of pri-let-7a-1 by KHSRP was normalized with total pri-let-7a-1 in 293T cells (left panel). The expression level of mature let-7a was analyzed by qRT-PCR (middle panel) and the immunoprecipitation efficiency was assessed by Western blotting (right panel). **c-d** The SUMO-site mutation K87R of KHSRP increases its interaction with pri-let-7a-1 or pri-let-7a-3 and mature miRNA production. 293 T cells transfected with CD513B-pri-let-7a-1 (**c**) or CD513B-pri-let-7a-3 (**d**) and Flag-KHSRP-WT or Flag-KHSRP-K87R were lysed for RIP with anti-Flag antibody, and then treated with Trizol, followed by qRT-PCR for pri-let-7a-1 or pri-let-7a-3. The relative recruitment fold of pri-let-7a-1 or pri-let-7a-3 by KHSRP was normalized (left panel). The expression levels of mature let-7a were analyzed by qRT-PCR (right panel) and the immunoprecipitation efficiency was assessed by Western blotting (bottom panel)

Since the KHSRP activity in miRNA biogenesis is promoted by the kinase ATM mediated- phosphorylation through the enhanced interaction of KHSRP and *TL-G-Rich* pri-miRNAs [8], we questioned whether SUMOylation of KHSRP influences the interaction between KHSRP and pri-miRNAs. To this end, we firstly predicted the secondary structure of pri-miRNAs with the RNAstructure V5.3 software, which is based on the minimum free energy principle according to the RNA primary sequence [45]. Most of these pri-miRNAs, whose mature miRNAs were upregulated by re-expression of KHSRP-K87R compared to those of re-expression of KHSRP-WT in DU145-shKHSRP stable cell lines (Fig. 4d; Additional file 10: Table S4), harbored short G-rich stretches in their terminal loops. For instances, as like pri-let-7a-1 [9, 10], the secondary structures of pri-miRNAs including pri-let-7a-3, pri-let-7g, pri-let-7i, pri-let-7e, pri-miR-98 and pri-miR-182 contained G-rich stretches (Fig. 6a).

Next, we performed the RIP (RNA-immunoprecipitation) assays [20, 26] to confirm the effect of SUMOylation on the interaction between KHSRP and pri-miRNAs. 293T cells transfected with pri-let-7a-1 and Flag-tagged KHSRPΔN, or SUMO1-KHSRPΔN were lysed for RIP (Fig. 6b, right panel) and followed by qRT-PCR analysis, showing that the interaction between pri-let-7a-1 and SUMO1-KHSRPΔN was decreased and consequently the mature let-7a biogenesis was downregulated compared to that of KHSRPΔN (Fig. 6b). Consistently with this, by using the same RIP assay we also found that the binding of pri-let-7a-1 or pri-let-7a-3 with KHSRP-K87R was significantly higher compared to that with KHSRP-WT, and accordingly the biogenesis of mature let-7a was much more in KHSRP-K87R than in KHSRP-WT (Fig. 6c-d). Above results demonstrate that SUMOylation of KHSRP inhibits its interactions with *TL-G-Rich* miRNAs precursors, consequently reducing the biogenesis of those miRNAs.

## Discussion

We recently reported that SUMOylation is involved in the regulation of miRNA pathways. TARBP2 SUMOylation does not influence the biogenesis of mature miRNAs, but it enhances the gene-silencing efficiency of miRNAs and suppresses tumor progression [26]. DGCR8 SUMOylation majorly occurs at two sites K707 and K259. K707-SUMOylation of DGCR8 increases its affinity with pri-miRNAs and directs the function of pri-miRNAs in oncogenic gene silencing, which promotes tumorigenesis and tumor cell migration [20]. However, K259-SUMOylation of DGCR8 promoted by the tumor suppressor p14ARF mainly maintains its nuclear localization to function as a partner of Drosha in the MC complex, which prevents the aberrant miRNA biogenesis and exerts its tumor-suppressive function [27]. KHSRP exists in the Drosha-DGCR8 complex as a single stranded RNA binding protein and promotes a subset of miRNAs biogenesis [7–10]. Here we demonstrated for the first time that SUMOylation of KHSRP regulates the *TL-G-Rich* miRNA biogenesis (Fig. 4c-e; Additional file 8: Table S2, Additional file 9: TableS3 and Additional file 10: Table S4), which is a novel function of SUMOylation in the miRNA pathways.

We identified that KHSRP was modified by SUMO1 at the major site K87 (Figs. 1, 2 and Additional file 2: Fig. S2, Additional file 3: Fig. S3) adjacent to its nuclear localization signal or sequence (NLS) of KHSRP, which suggested that SUMOylation is involved in the regulation of nucleocytoplasmic transport. In most cases, SUMOylation promotes the nuclear import of target proteins, such as SUMOylation of RanGAP1, ZIC3 and JAK2 [22, 23, 43, 44]. But interestingly, in this study we found that SUMOylation of KHSRP promotes its nuclear export (Fig. 5), as like SUMOylation of p53 [24, 25]. SUMO1-KHSRP gene fusion mimicking SUMOylated status increased the cytoplasmic localization (Fig. 5c-d), but we could not exclude the possibility that SUMO1 fusion at the N terminus might affect the nuclear localization. However the cytoplasmic localization of KHSRP was increased by knockdown of SENP1, or under hypoxia environment, or stimulation by LY294002, for high-SUMOylation status (Fig. 5e-f, Additional file 13: Fig. S9 and Additional file 14: Fig. S10), and by co-expression of SUMO1 (Fig. 5b), which was similar to the translocation pattern of the NLS-deleted KHSRP (Fig. 5d). In agreement with these, the SUMO-site mutant KHSRP-K87R existed almost in the nucleus (Fig. 5a-b). Thus, we come to a conclusion that the translocation of KHSRP from the nucleus to the cytoplasm is partially controlled by SUMOylation.

KHSRP can recognize and bind to *TL-G-Rich* the terminal loop (TL) of a subset of miRNA precusors which harbor short G-rich sequences, and promote their processing to mature miRNAs [7, 9, 10]. Among these miRNAs, the let-7 family, which are important tumor suppressor miRNAs [46, 47], are the most classic examples of KHSRP positively regulating miRNA biogenesis [7, 10]. Indeed, 151 miRNAs including let-7 family were down-regulated due to KHSRP knockdown (Fig. 4c; Additional file 9: Table S3). We observed that SUMOylation promotes translocation of KHSRP from the nucleus to the cytoplasm, which probably explained why the binding of Drosha-DGCR8 complex with KHSRP-K87R was increased compared to that with KHSRP-WT (Fig. 4a-b). As expectedly, 51 miRNAs including let-7i, let-7g, let-7e and miR-98 were up-regulated by the mutant KHSRP-K87R compared with by KHSRP-WT (Fig. 4c-d; Additional file 10: Table S4). Moreover, by using the RNAstructure software, we analyzed the secondary structures to show short G-rich stretches in the terminal loop of these pri-miRNAs (Additional file 10: Table S4), for instances, pri-let-7a-1, pri-let-7a-3, pri-let-7e, pri-let-7g, pri-let-7i, miR-98 and pri-miR-182 (Fig. 6a). The RIP and qPCR assays revealed that the fusion of SUMO1 to the KHSRP obviously inhibited its interaction with pri-let-7a-1 thus leading to the decrease of mature let-7a (Fig. 6b), whereas the SUMO-site mutant KHSRP-K87R extremely enhanced the interaction between KHSRP and pri-let-7a-1 or pri-let-7a-3 thereby resulting in the increase of mature let-7a (Fig. 6c-d). Therefore, besides altering its translocation, SUMOylation of KHSRP directly interfered its binding to pri-miRNAs, which also contributed to KHSRP SUMOylation inhibiting the biogenesis of a subset of miRNAs. The third KH domain (KH3) of KHSRP recognizes short G-rich sequences in the pre-let-7 terminal loop and dominates the interaction [10], but it is not clear whether SUMOylation of the protein influences the formation of the KH3-pri-miRNA complex.

The miRNA pathways are involved in diverse physiological and pathological processes including cancer. Impaired microRNA processing enhances cellular transformation and tumorigenesis by knockdown of the components of the miRNA processing machinery such as Dicer and Drosha [48]. TARBP2 recruiting Dicer to Ago2 constitutes an RNA-induced silencing complex (RISC)-loading complex (RLC) for miRNA processing and gene silencing [26, 49], and recently we discovered SUMOylation of TARBP2 plays roles in suppression of tumor growth and tumor cell migration by regulating miRNA efficiency rather than influencing the mature miRNA production [26]. DGCR8 is the most important binding partner protein of Drosha, and most recently we found that DGCR8 can be SUMOylated at two sites with reverse functions of each other, showing that SUMOylation at K707 promotes tumorigenesis [20] while SUMOylation at K259 suppresses tumorigenesis [27]. KHSRP is also a very important component of the Drosha/DGCR8 complex, and here we for the first time found that KHSRP SUMOylation was also linked to tumorigenesis and cancer progression. The abilities of soft-agar colony formation, migration, invasion and xenografted tumor growth were increased when KHSRP stably knockdown in DU145 cells (Fig. 3a-e), which was attributed to downregulation of *TL-G-Rich* miRNAs (Fig. 4c; Additional file 9: Table S3). This suggested that KHSRP plays a key role in tumor-suppression. Compared with KHSRP-WT, re-expression of KHSRP-K87R into stable cell line DU145-shKHSRP, a subset of miRNAs such as let-7 family were upregulated (Fig. 4d-e; Additional file 10: Table S4) and consequently the tumor-suppressive capabilities were enhanced (Fig. 3a-e).

## Conclusions

**Fig. 7:**
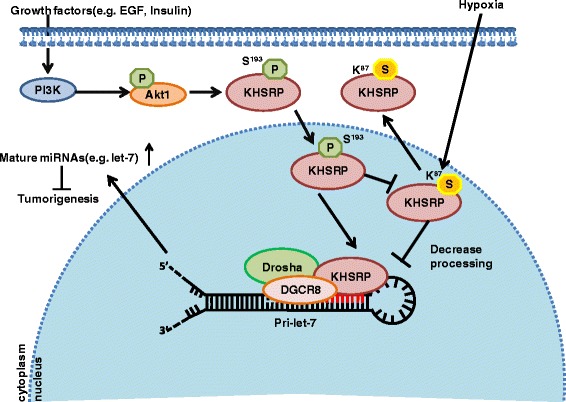
A model for SUMOylation of KHSRP inhibiting miRNA biogenesis. In brief, EGF or insulin can block the K87-SUMO1 modification on KHSRP through phosphorylation of KHSRP by PI3K/AKT pathway to promote its interaction with Drosha/DGCR8 and pri-miRNAs, sequentially affects a subset of miRNAs biogenesis. Reversely, KHSRP SUMO1 modification is enhanced in hypoxia condition, which leads to its cytoplasm shifting and alters the miRNA biogenesis profile to promote tumor processes. Thereby, K87-SUMO1 modification on KHSRP may play a critical function in tumorgenesis through regulating the microprocessor procedure of partial specific pri-miRNAs to generate mature miRNAs

Finally as summarized in Fig. 7, we discovered a novel mechanism underlying SUMOylation of KHSRP regulating the production of some special miRNAs. KHSRP SUMOylation was inhibited by growth factors such as EGF and insulin, which activated its phosphorylation to impede SUMOylation. In contrast, microenvironmental hypoxia and LY294002 enhanced KHSRP SUMOylation. SUMOylation of KHSRP might suppress its binding with the pri-miRNAs and Drosha-DGCR8 complex and promote its translocation from the nucleus to cytoplasm. As a result of the above orchestrating, the pre-miRNA processing from pri-miRNA harboring short G-rich stretches in the terminal loop was impaired, thus leading to the decrease of a subset of mature miRNAs, especially like tumor suppressive miRNAs let-7 family. Our data indicate that manipulation of the SUMOylation/miRNA pathway may represent an innovative strategy for a better cancar therapy.

## Methods

### Cell cultures and transfections

Human embryonic kidney 293T, 293FT, HeLa and prostate cancer DU145 cell lines were cultured in Dulbecco’s modified Eagle’s medium (DMEM, obtained from Hyclone) containing 10% fetal calf serum (Biowest, Kansas, MO, USA), 1% penicillin and streptomycin (Invitrogen). All cell lines were cultured at 37 °C in a 5% CO_2_ humidified incubator. Cell transfection was performed using Lipofectamine 2000 (Invitrogen).

### Reagents and antibodies

Antibody against KHSRP (#A302–021) was from Bethyl Laboratories, Inc. (Montgomery, UK). Tubulin-Alpha mouse Mcab (#66031–1-Ig), His-Tag mouse antibody (#66005–1-Ig), GST mouse antibody (#66001–1-Ig) were from Proteintech™. GAPDH (#ab37168), SUMO1 (Y299, #ab32058) antibodies were from Abcam. Mouse antibodies against Flag (M2, #F1804), HA (16B12, #MMS-101P) were from Sigma. Rabbit antibodies against HA (Y-11, #sc805), Ubc9 (H-81, #sc-10,759) and mouse antibody against Lamin B1 (8D1, #sc-56,144) were from Santa Cruz Biotech. Rabbit antibody against SUMO1 (C9H1, #4940S) was from Cell Signal Technology. Alexa Fluor® 568 (Rabbit, #A11011), 488 (Rabbit, #A11008), 488 (Mouse, #A11001) were from Invitrogen.

Protein G Plus/Protein A agarose suspension (#IP05) were purchased from Calbiochem. Puromycin (#P8833), hydrogen peroxide (H_2_O_2_) solution (#H1009), insulin (#I-5500), LY294002 (#L9908) and EGF (#E9644) were from Sigma.

### Plasmids

The pEGFP-6xHis-FLKHSRP plasmid was from Addgene [11]. The FLKHSRP (full-length KHSRP) was amplified by PCR, and subcloned into the pEF-5HA vector with *EcoR*I and *Xba*I sites, the pCMV-Tag2B vector with *EcoR*I and *Xho*I sites, the pGEX-4T-1 vector with *EcoR*I and *Xho*I sites, respectively. Point mutations (K87R, K359R, K628R, S193A, S193D, K244R, K251R, K435R, K473R and K494R) and nuclear localization sequence (NLS) deletion mutant of KHSRP (KHSRPΔNLS) were carried out by using KOD-plus-mutagenesis Kit (TOYOBO) according to the manual. The pEF-5HA-KHSRPΔN (N-terminal aa 1–67 was deleted) was amplified from Flag-KHSRPΔN which was kindly provided by Michael G. Rosenfeld’s Lab [7] by using KOD plus (TOYOBO) and subcloned into the pEF-5HA vector with *EcoR*I and *Xba*I sites. The KHSRP cDNA was amplified from pCMV-Tag2B-KHSRP with the primer containing KOZAK (GCCACC) and HA tag sequence, and then subcloned into the lentiviral vector CD513B (System Biosciences) carrying EGFP and puromycin genes. The shRNA anti-KHSRP were obtained from Sigma and sub-cloned into pLKO.1 vector.

Flag-SUMO1-KHSRPΔN plasmid was constructed by two steps. Firstly, KHSRPΔN was amplified from pEF-5HA-KHSRPΔN by using KOD plus (TOYOBO) with 10% DMSO, and then subcloned into the pCMV-Tag2B vector with *Sal*I and *Xho*I sites. Secondly, SUMO1 (amino acids 2–96) was amplified from His-SUMO1 and inserted into of pCMV-Tag2B-KHSRPΔN with *Hind*III and *Sal*I sites. All above plasmids were verified by sequencing. Primers used for constructions are shown in Additional file 16: Table S5.

### SUMOylation assays

(A) SUMOylation of KHSRP was analyzed in 293T cells by using the method of Ni^2+^-NTA beads with His-tagged SUMO1, as described previously [29, 50, 51]. (B) In vitro SUMOylation assay in *E.coli* system with pE1E2SUMO1 was performed as previously described [20, 29]. Briefly, pGEX-4T-1-KHSRP-WT was co-expressed with or without pE1E2SUMO1 plasmid in *E.coli* BL21 (DE3) respectively, and then lysed by using B-PER Protein Extraction Reagent (#78248, Thermo Fisher, USA) and incubated with Glutathione sepharose 4B (GE healthcare) at 4 °C overnight. The beads bound proteins were washed for three times with lysis buffer, and subjected to Western-bot for analysis of SUMO1-modified GST-KHSRP. (C). The method of immunoprecipitation (IP) was also used to detect the SUMO1 modification of endogenous KHSRP. Briefly, 293T cells were lysed in NEM-RIPA buffer (50 mM Tris–HCl pH 7.4, 150 mM NaCl, 1% NP-40, 20 mM N-ethylmaleimide and one complete protease inhibitor cocktail). Cell lysates (1 mg) were used for immunoprecipitation. To detect the endogenous SUMO1-KHSRP in 293T cells, 5 μl of KHSRP antibody or normal IgG (as a control) was used for immunoprecipitation. Tissues were lysed in NEM-RIPA with 0.1% SDS and 5 mM EDTA, which was according to our previous study [28] and SUMO1 antibody was used for immunoprecipitation.

### Nuclear/cytosol fractionation assay

Nuclear and cytosolic fractions were extracted by Nuclear/Cytosol fractionation kit according to the manual (Nuclear/Cytosol Fractionation Kit, Catalog #K266, BioVision, BioVision Incorporated, Milpitas, CA 95035 USA). 3 × 10^6^ of HeLa cells were harvested. One-tenth of these cells were harvested by SDS lysis as the Input for the protein expression by Western-blot, and nine-tenth of cells were extracted by Nuclear/Cytosol fractionation kit. One-fifth of both nuclear and cytoplasmic fraction was used for Western-blotting with indicated antibody.

### Immunofluorescent staining

Immunofluorescent staining was performed as described previously [28, 50]. Briefly, HeLa cells were seeded into the Poly-Lysine coated slides overnight, and then transfected with plasmids expressing Flag-KHSRPΔN, Flag-SUMO1-KHSRPΔN or Flag-KHSRPΔNLS, respectively. After 48 h, cells were fixed with 4% paraformaldehyde for 30 min and then permeabilized with 0.2% Triton X-100 for 1 h, and then incubated in the primary antibody anti-Flag (M2) (dilution 1:800) at 4 °C overnight. Cells were washed three times with PBS and then incubated in the secondary antibody (Alexa 488 anti mouse, dilution 1:500) in blocking solution for 2 h. The cells were then washed five times with PBS. DAPI (4′,6-diamidino-2-phenylindole) was added to visualize the nucleus. Images were taken with Nikon microscope.

In another experiment, HeLa cells were transfected with HA-KHSRP-WT or HA-KHSRP-K87R with GFP-SUMO1. Cells were incubated with the primary antibody anti-HA (Rabbit, dilution 1:250), with the secondary antibody Alexa 568 anti rabbit (dilution 1:500). DAPI staining was to visualize the nucleus. The images of DAPI and anti-HA were merged. The green signals indicating the expression of GFP-SUMO1 were directly observed and taken by Nikon microscope.

To detect the endogenous KHSRP in HeLa cells, KHSRP antibody (Rabbit, dilution 1:500) was used as primary antibody and Alexa 488 anti-rabbit antibody (dilution 1:500) was used as secondary antibody.

### Co-immunoprecipitation (co-IP)

293T cells transfected with HA-Drosha and Flag-KHSRP-WT or -K87R, or cells transfected with Flag-DGCR8 and HA-KHSRP-WT or -K87R respectively were lysed in RIPA buffer (50 mM Tris-HCl pH = 7.4, 150 mM NaCl, 1% NP40, 10% glycerol and a complete protease inhibitor cocktail (Roche)). 1 mg of total extracted proteins were incubated with 25 μl of protein A/G agarose and 2 μg of anti-Flag antibody at 4 °C overnight. Then the beads were washed with RIPA buffer for three times, and followed by Western blot analysis.

### High-throughput miRNA sequencing

The method of high-throughput sequencing was previously described [52]. Briefly, total RNAs from DU145 stable cell lines were extracted by Trizol (Life Technologies, Carlsbad, CA). The RNAseq library of miRNA was prepared using NEBNext® Multiplex Small RNA Library Prep Set for Illumina (NEB, Beverly, MA). Modified RNAs were reversely transcribed and then PCR amplified with specific primers corresponding to the adapters. The amplified products were resolved in 6% PAGE and the bands corresponding to ~140 bp were isolated. The libraries were quality controlled with a Bioanalyzer 2100 (Agilent, Santa Clara, CA) and sequenced by Nextseq 500 (Illumina, San Diego, CA) on a 75 bp single-end run.

### RNAseq analysis

The raw sequencing reads from the small RNA libraries were mapped to the human genome (hg19) using the mapper.pl script included in miRDeep2 program with the following parameters: -l,-k,-e,-h,-j,-m. This clipped the adapter sequence from each read while keeping only reads no shorter than 18 nt. Specifically, the mapper.pl package takes Bowtie2 as the mapping engine and generated a collapsed set of non-redundant reads along with the mapped genomic locations. Then the quantifier.pl script was used to calculate the expression level of known microRNAs from the annotation of miRBase (Release 21). Additionally, the normalized abundance (RPM, Reads Per Million) for each known microRNAs was calculated as following:

$$ {RPM}_i=1000,000\times \frac{R_i}{N_j} $$

Where Ri represents the count of reads mapped to the genomic region of a given microRNA i. And Nj represents the total count of reads in the small RNA library of sample j.

### RNA immunoprecipitation assay (RIP)

The RNA immunoprecipitation assay (RIP) was performed as previously described [20, 26]. Briefly, 48 h after transfection with the indicated plasmids, one-tenth of these cells in 10-cm plate cultured were reserved as the Input for qRT-PCR analysis, and nine-tenth of cells were lysed in RIP buffer (150 mM NaCl, 50 mM Tris-HCl pH 7.4, 1% NP40, 1 mM DTT, 100 units/ml RNase inhibitor (Fermentas), 400 μM VRC (New England BioLabs), Protease inhibitor cocktail (Roche)). After incubated on ice for 1 h, 1/50 of lysates were used for Western blot to examine the expression of KHSRP, the others were incubated with 40 μl of protein A/G agarose and 4 μg of anti-Flag antibody at 4 °C overnight. After immunoprecipitation, the beads were washed with RIP-lysis buffer for three times and then 1/10 of the beads were immunoblotted for the efficiency analysis of immunoprecipitation. The remaining beads were used to extract RNA by Trizol reagent (Invitrogen).

### qRT-PCR analysis

Quantitative real-time PCR was performed with SYBR® Green PCR Master Mix (#4309155, Applied Biosystems, USA) to analyze the fold changes of pri-miRNAs immunoprecipitated by KHSRP and mature miRNAs biogenesis. GAPDH and U6 levels were used for normalization of pri-miRNAs and mature miRNAs PCR and the pri-miRNAs immunoprecipitated by KHSRP was normalized by Input of pri-miRNAs.

### Soft-agar colony forming assay

The soft-agar colony forming assay was performed as described previously [20, 26, 50]. Briefly, this assay was performed in 6-well plates with a base of 2 ml of medium containing 5% FBS with 0.6% Bacto agar (Amresco). Cells were seeded in 2 ml of medium containing 5% FBS with 0.35% agar at 2 × 10^3^ (for DU145 cells) cells/well and layered onto the base. 2 ml of DMEM with 10% FBS was covered on the top of agar gel. The photographs of colonies growing in the plates were taken at day 21. The number of colonies was scored by photoshop CS5.

### Migration assay by RTCA-DP

The procedure was carried out as previously described [20, 26]. Briefly, 4 × 10^4^ of each of serum-starved DU145 stable cells were suspended in 100 μl of serum-free medium, and then cell suspension was added into the pre-equilibrated upper chamber of CIM-plate. The lower chamber was filled with 160 μl of complete DMEM containing 10% FBS. The kinetic cell index of migration was recorded every 15 min for 24 h and then calculated by RTCA software v1.2 (Roche Applied Science).

### Vasculogenic mimicry (VM) assay

The vasculogenic mimicry experiment of DU145 stable cells were carried out using μ-Slide Angiogenesis Kit (IBIDI) according to the procedure as previously described [39, 53]. DU145 cells at the density of 5 × 10^3^ in 50 μl of growth medium were plated in each well pre-coated with matrix™ (Millipore). Pictures were taken with Nikon microscope 20 h later.

### Three-dimensional (3D) cell culture assay

The 3D cell culture assay was performed as described before [39]. Generally, 5 μl of 3D matrix™ (Millipore) and 5 μl of cell solution (2 × 10^3^ cells) were mixed and added into the inner well of μ-Slides (IBIDI) and covered with complete cell culture medium. The μ-slides were incubated at 37 °C and 5% CO_2_ cell incubator for 7 days and pictures were taken with Nikon microscope.

### Xenograft tumor model

The experiment of xenograft tumor model was conducted as previously described [20, 50]. Stable DU145 cell lines (2.5 × 10^6^) were injected subcutaneously into 5-week-old male BALB/c nude mice (*n* = 5) individually. 15 days after injection, the tumors were measured every 6 days. All mice were sacrificed at 35 days and the tumors were dissected, photographed and weighed. All animal studies were conducted with the approval and guidance of Shanghai Jiao Tong University Medical Animal Ethics Committees.

### Statistical analysis

Experiments were performed at least three times, and representative results were shown. All data are presented as means ± s.e.m. for qPCR, RTCA migration, mouse xenograft model and soft agar colony forming assay. Statistical analysis was calculated with Microsoft Excel analysis tools. Differences between individual groups are analyzed using the t-test (two-tailed and unpaired) with triplicate or quadruplicate sets. A value of *P* < 0.05 was considered statistically significant and *P*-value <0.05 was marked with (*), < 0.01 with (**) or <0.001 with (***). TCGA data was analyzed by Pearson Correlation method by the program SPSS 22.

## Additional files

Additional file 1: **Fig. S1.** Endogenous KHSRP can be modified by SUMO1. 293T cells were lysed by RIPA buffer. Co-IP experiment was used to detect the interaction between KHSRP and SUMO1. The proteins was immunoprecipitated by anti-KHSRP antibody. Western blotting was conducted with anti-SUMO1 antibody and the same membrane was detected with anti-KHSRP antibody after stripping (PDF 686 kb)

Additional file 2: **Fig. S2.** The SUMOylation sites according to SUMOplot™. SUMOylation sites of human KHSRP protein were predicted by the program of Abgent SUMOplot™ (http://www.abgent.com/sumoplot). K87 shows the second highest score (0.62) (PDF 1081 kb)

Additional file 3: **Fig. S3.** K87 is the main site of SUMO1 modification of KHSRP. HA-KHSRP WT or -K87R, or -K244R, or -K251R, or -K435R, or -K473R, or -K494R were co-transfected with His-SUMO1 into 293T cells. Cells were lysed 48 h after transfection and Ni^2+^-NTA resin pull down was performed to detect SUMO1 modification of HA-KHSRP (PDF 890 kb)

Additional file 4: **Fig. S4.** Expression of endogenous and exogenous KHSRP in DU145 stable cell lines. Endogenous KHSRP was stably knocked down in DU145 cell and then empty vector, HA-KHSRP WT, or –K87R was re-introduced. Endogenous and exogenous KHSRP expression was verified by western blot (PDF 377 kb)

Additional file 5: **Fig. S5.** The xenografted tumor volume of DU145 stable cell lines in nude mice. The DU145 stable cell lines were injected subcutaneously into male BALB/c nude mice. 5 male BALB/c nude mice were injected subcutaneously with stable DU145 cell lines (2.5 × 10^6^ cells/each) expressing the shRNA control vector in the left back and shKHSRP in the right back, respectively. Another 5 male BALB/c nude mice were injected subcutaneously with stable DU145 cell lines expressing shKHSRP-KHSRP WT in the left back and shKHSRP-KHSRP K87R in the right back, respectively. The sizes of tumors were measured at 15, 21, 27 and 32 days after injection (PDF 546 kb)

Additional file 6: **Table S1.** The transcript expressions extracted from TCGA database is presented in the normalized FPKM (Fragments Per Kilobase of transcript per Milllion fragments mapped) (PDF 92 kb)

Additional file 7: **Fig. S6.** Endogenous SUMO1 modification of KHSRP in clinical cancers. Tumors (T) and paracancerous tissues (P) of gastric cancer (GC) and colorectal cancer (CRC) were lysed in NEM-RIPA buffer and then the proteins were immunoprecipitated by anti-SUMO1 antibody and Western-blotting with indicated antibodies (PDF 458 kb)

Additional file 8: **Table S2.** MiRNAs expression in DU145 shRNA Ctrl and shKHSRP stabel cell lines (PDF 74 kb)

Additional file 9: **Table S3.** A subset of miRNAs biogenesis was downregulated in DU145 shKHSRP stable cell lines (PDF 49 kb)

Additional file 10: **Table S4.** KHSRP K87R promotes a subset of miRNAs biogenesis in DU145 stable cell lines (PDF 76 kb)

Additional file 11: **Fig. S7.** SUMO1 modification promotes KHSRP cytoplasmic translocation. The additional representative images of cells showing cytoplasmic HA-KHSRP-WT was presented. Scale bar, 25 μm (PDF 505 kb)

Additional file 12: **Fig. S8.** Expression of Flag-KHSRPΔN and Flag-SUMO1-KHSRPΔN in HeLa cells. HeLa cells were transfected with Flag-KHSRPΔN and Flag-SUMO1-KHSRPΔN. 48 h after transfection, 1/10 HeLa cells were harvested with SDS buffer for Input and 9/10 HeLa cells were harvested with the nuclear/cytosol fractionation kit. The expression of Flag-KHSRPΔN or Flag-SUMO1-KHSRPΔN was determined by Western blotting (PDF 333 kb)

Additional file 13: **Fig. S9.** Hypoxia promotes KHSRP cytoplasmic localization. HeLa cells were cultured in 1% oxygen condition (hypoxia) for 0, 12 h before cells were harvested. (A) Nuclear and cytosolic fractions were extracted by the Nuclear/Cytosol fractionation kit. (B) Endogenous KHSRP was stained with the primary antibody anti-KHSRP (Rabbit), and then with the second antibody of Alexa Fluor 488 anti-rabbit. DAPI staining was to visualize the nucleus. All the images were taken by Nikon microscope, scale bar =25 μm (PDF 602 kb)

Additional file 14: **Fig. S10.** Hypoxia promotes KHSRP cytoplasmic localization. HeLa cells were stimulated by LY294002 (25 μM) for 0, 16 h before cells were harvested. (A) Nuclear and cytosolic fractions were extracted by the Nuclear/Cytosol fractionation kit. (B) Endogenous KHSRP was stained with the primary antibody anti-KHSRP (Rabbit), and then with the second antibody of Alexa Fluor 488 anti-rabbit. DAPI staining was to visualize the nucleus. All the images were taken by Nikon microscope, scale bar =25 μm (PDF 549 kb)

Additional file 15: **Fig. S11.** Expression of endogenous SENP1 and Ubc9 in HeLa shSENP1 and shUbc9 stable cell lines. Endogenous SENP1 and Ubc9 was stably knocked down in HeLa cells, respectively. Endogenous SENP1 and Ubc9 expression was verified by western blot, respectively. We chose the third HeLa shSENP1 stable cell line marked with asterisk for experiment (PDF 650 kb)

Additional file 16: **Table S5.** All primers or oligonucleotides used in this study (PDF 245 kb)

## Abbreviations

EGF: Epidermal growth factor
KHSRP: hnRNP K homology (KH)-type splicing regulatory protein
NLS: Nuclear localization sequence
RTCA: Real-time cell analyzer
SUMO: Small ubiquitin-like modifier
VM: Vasculogenic mimicry
