# Supplementary figures and images for: SUMO1 modification of KHSRP regulates tumorigenesis by preventing the TL-G-Rich miRNA biogenesis
Source: Mol Cancer. 2017 Oct 11;16:157. doi: 10.1186/s12943-017-0724-6 (PMC5637259; doi:10.1186/s12943-017-0724-6)

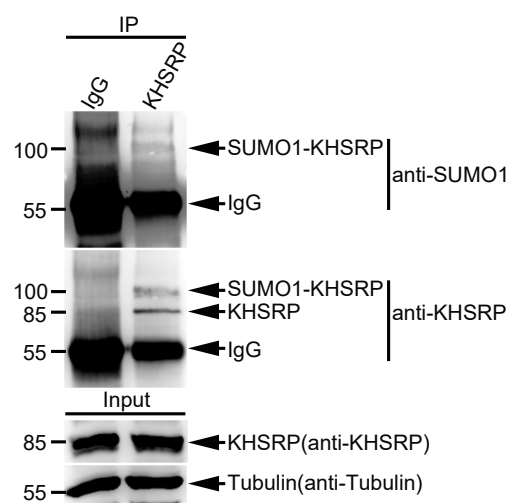

Supplement: Supplementary file 1 — Fig. S1. Endogenous KHSRP can be modified by SUMO1. 293T cells were lysed by RIPA buffer. Co-IP experiment was used to detect the interaction between KHSRP and SUMO1. The proteins was immunoprecipitated by anti-KHSRP antibody. Western blotting was conducted with anti-SUMO1 antibody and the same membrane was detected with anti-KHSRP antibody after stripping (PDF 686 kb) [file 12943_2017_724_MOESM1_ESM.pdf]

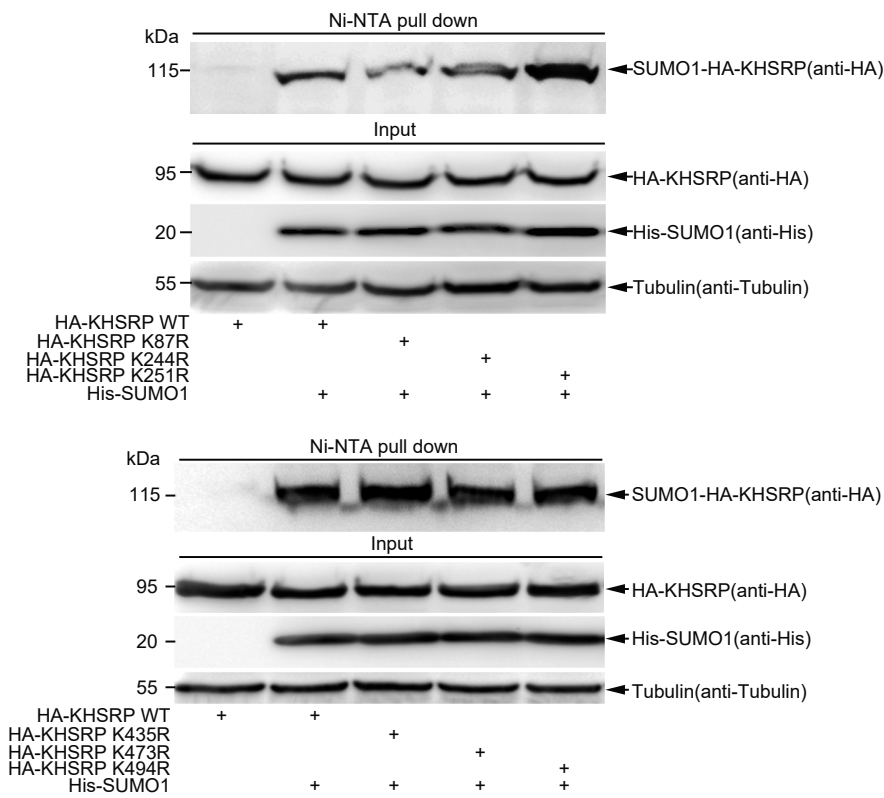

Supplement: Supplementary file 3 — Fig. S3. K87 is the main site of SUMO1 modification of KHSRP. HA-KHSRP WT or -K87R, or -K244R, or -K251R, or -K435R, or -K473R, or -K494R were co-transfected with His-SUMO1 into 293T cells. Cells were lysed 48 h after transfection and Ni2+-NTA resin pull down was performed to detect SUMO1 modification of HA-KHSRP (PDF 890 kb) [file 12943_2017_724_MOESM3_ESM.pdf]

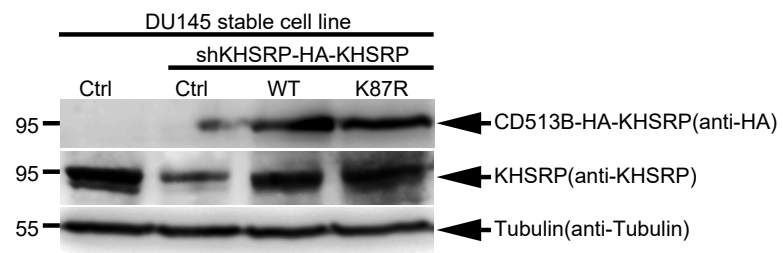

Supplement: Supplementary file 4 — Fig. S4. Expression of endogenous and exogenous KHSRP in DU145 stable cell lines. Endogenous KHSRP was stably knocked down in DU145 cell and then empty vector, HA-KHSRP WT, or –K87R was re-introduced. Endogenous and exogenous KHSRP expression was verified by western blot (PDF 377 kb) [file 12943_2017_724_MOESM4_ESM.pdf]

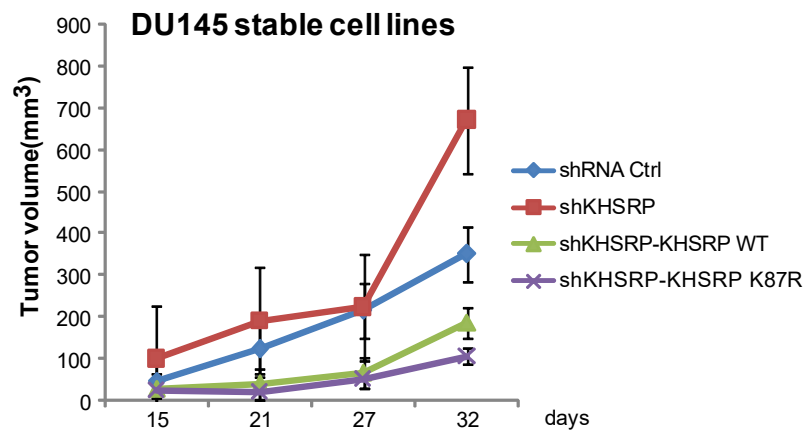

Supplement: Supplementary file 5 — Fig. S5. The xenografted tumor volume of DU145 stable cell lines in nude mice. The DU145 stable cell lines were injected subcutaneously into male BALB/c nude mice. 5 male BALB/c nude mice were injected subcutaneously with stable DU145 cell lines (2.5 × 10^6^ cells/each) expressing the shRNA control vector in the left back and shKHSRP in the right back, respectively. Another 5 male BALB/c nude mice were injected subcutaneously with stable DU145 cell lines expressing shKHSRP-KHSRP WT in the left back and shKHSRP-KHSRP K87R in the right back, respectively. The sizes of tumors were measured at 15, 21, 27 and 32 days after injection (PDF 546 kb) [file 12943_2017_724_MOESM5_ESM.pdf]

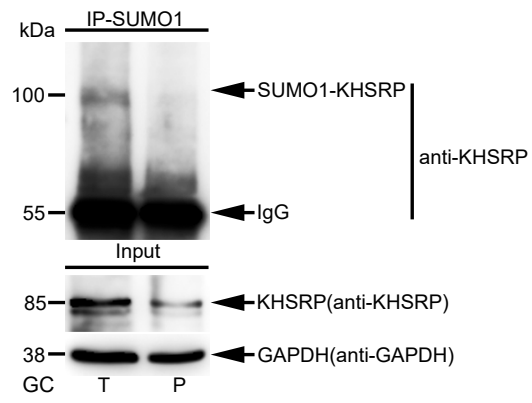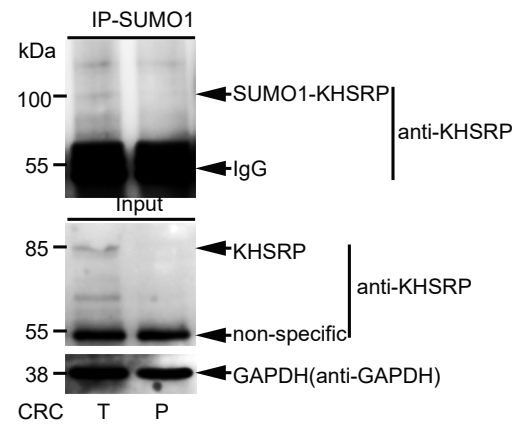

Supplement: Supplementary file 7 — Fig. S6. Endogenous SUMO1 modification of KHSRP in clinical cancers. Tumors (T) and paracancerous tissues (P) of gastric cancer (GC) and colorectal cancer (CRC) were lysed in NEM-RIPA buffer and then the proteins were immunoprecipitated by anti-SUMO1 antibody and Western-blotting with indicated antibodies (PDF 458 kb) [file 12943_2017_724_MOESM7_ESM.pdf]

F

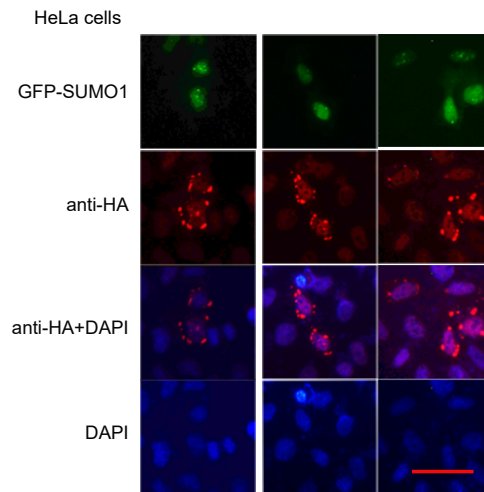

Supplement: Supplementary file 11 — Fig. S7. SUMO1 modification promotes KHSRP cytoplasmic translocation. The additional representative images of cells showing cytoplasmic HA-KHSRP-WT was presented. Scale bar, 25 μm (PDF 505 kb) [file 12943_2017_724_MOESM11_ESM.pdf]

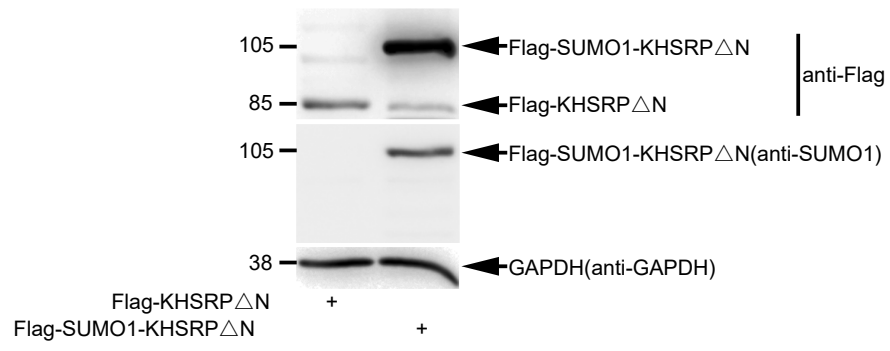

Supplement: Supplementary file 12 — Fig. S8. Expression of Flag-KHSRPΔN and Flag-SUMO1-KHSRPΔN in HeLa cells. HeLa cells were transfected with Flag-KHSRPΔN and Flag-SUMO1-KHSRPΔN. 48 h after transfection, 1/10 HeLa cells were harvested with SDS buffer for Input and 9/10 HeLa cells were harvested with the nuclear/cytosol fractionation kit. The expression of Flag-KHSRPΔN or Flag-SUMO1-KHSRPΔN was determined by Western blotting (PDF 333 kb) [file 12943_2017_724_MOESM12_ESM.pdf]

A

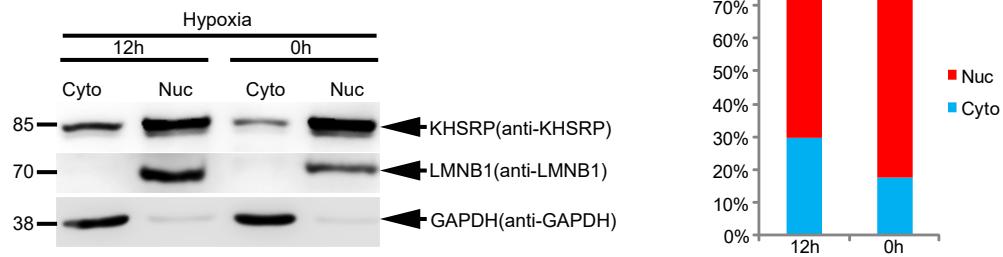

B

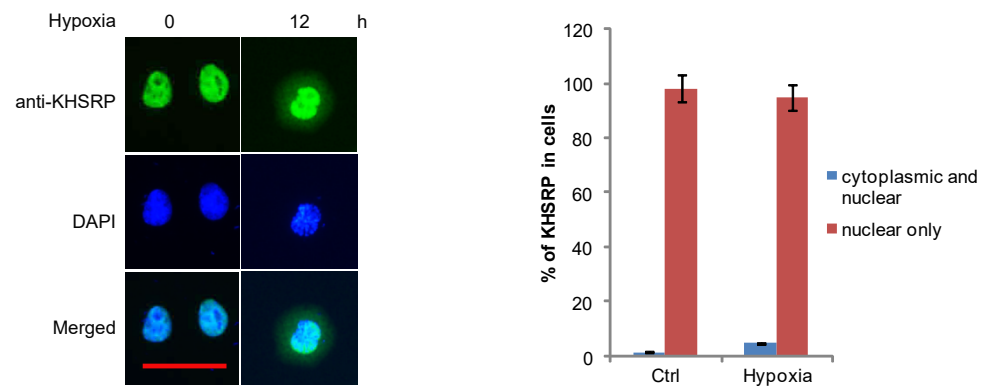

Supplement: Supplementary file 13 — Fig. S9. Hypoxia promotes KHSRP cytoplasmic localization. HeLa cells were cultured in 1% oxygen condition (hypoxia) for 0, 12 h before cells were harvested. (A) Nuclear and cytosolic fractions were extracted by the Nuclear/Cytosol fractionation kit. (B) Endogenous KHSRP was stained with the primary antibody anti-KHSRP (Rabbit), and then with the second antibody of Alexa Fluor 488 anti-rabbit. DAPI staining was to visualize the nucleus. All the images were taken by Nikon microscope, scale bar =25 μm (PDF 602 kb) [file 12943_2017_724_MOESM13_ESM.pdf]

A

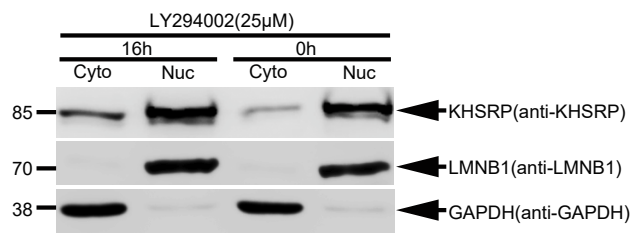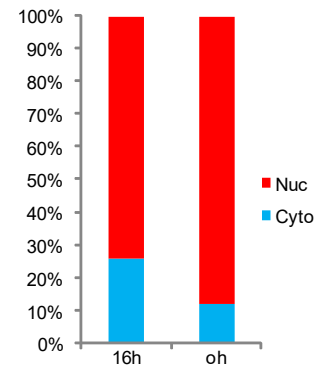

B

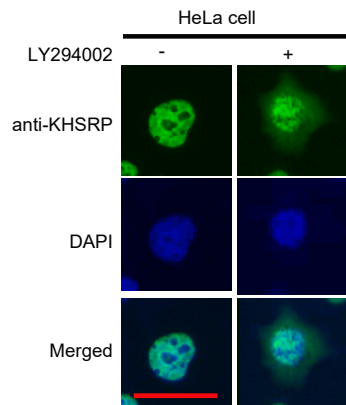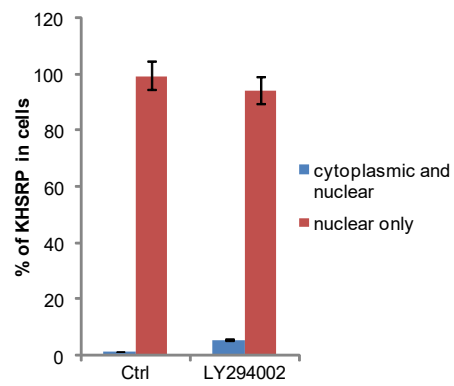

Supplement: Supplementary file 14 — Fig. S10. Hypoxia promotes KHSRP cytoplasmic localization. HeLa cells were stimulated by LY294002 (25 μM) for 0, 16 h before cells were harvested. (A) Nuclear and cytosolic fractions were extracted by the Nuclear/Cytosol fractionation kit. (B) Endogenous KHSRP was stained with the primary antibody anti-KHSRP (Rabbit), and then with the second antibody of Alexa Fluor 488 anti-rabbit. DAPI staining was to visualize the nucleus. All the images were taken by Nikon microscope, scale bar =25 μm (PDF 549 kb) [file 12943_2017_724_MOESM14_ESM.pdf]

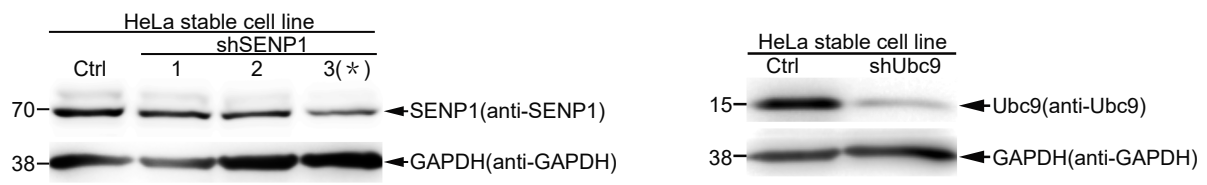

Supplement: Supplementary file 15 — Fig. S11. Expression of endogenous SENP1 and Ubc9 in HeLa shSENP1 and shUbc9 stable cell lines. Endogenous SENP1 and Ubc9 was stably knocked down in HeLa cells, respectively. Endogenous SENP1 and Ubc9 expression was verified by western blot, respectively. We chose the third HeLa shSENP1 stable cell line marked with asterisk for experiment (PDF 650 kb) [file 12943_2017_724_MOESM15_ESM.pdf]
