# Supplementary material for: SUMO1 modification of KHSRP regulates tumorigenesis by preventing the TL-G-Rich miRNA biogenesis
Source: Mol Cancer. 2017 Oct 11;16:157. doi: 10.1186/s12943-017-0724-6 (PMC5637259; doi:10.1186/s12943-017-0724-6)
Supplement: Supplementary file 2 — Fig. S2. The SUMOylation sites according to SUMOplot™. SUMOylation sites of human KHSRP protein were predicted by the program of Abgent SUMOplot™ (http://www.abgent.com/sumoplot). K87 shows the second highest score (0.62) (PDF 1081 kb) [file 12943_2017_724_MOESM2_ESM.pdf]

**Protein ID:**	gi|154355000|ref|NP_003676.2|
**Defintion:**	far upstream element-binding protein 2 [Homo sapiens]
**Length:**	711 aa

```
  1 MSDYSTGGPP PGPPPPAGGG GGAGGAGGGP PPGPPGAGDR GGGGPGGGGP
 51 GGGSAGGPSQ PPGGGGPGIR KDAFADAVQR ARQIAAKIGG DAATTVNNST
101 PDFGFGGQKR QLEDGDQPES KKLASQGDSI SSQLGPIHPP PRTSMTEEYR
151 VPDGMVGLII GRGGEQINKI QQDSGCKVQI SPDSGGLPER SVSLTGAPES
201 VQKAKMMLDD IVSRGRGGPP GQFHDNANGG QNGTVQEIMI PAGKAGLVIG
251 KGGETIKQLQ ERAGVKMILI QDGSQNTNVD KPLRIIGDPY KVQQACEMVM
301 DILRERDQGG FGDRNEYGSR IGGGIDVPVP RHSVGVVIGR SGEMIKKIQN
351 DAGVRIQFKQ DDGTGPEKIA HIMGPPDRCE HAARIINDLL QSLRSGPPGP
401 PGGPGMPPGG RGRGRGQGNW GPPGGEMTFS IPTHKCGLVI GRGGENVKAI
451 NQQTGAFVEI SRQLPPNGDP NFKLFIIRGS PQQIDHAKQL IEEKIEGPLC
501 PVGPGPGGPG PAGPMGPFNP GPFNQGPPGA PPHAGGPPPH QYPPQGWGNT
551 YPQWQPPAPH DPSKAAAAAA DPNAAWAAYY SHYYQQPPGP VPGPAPAPAA
601 PPAQGEPPQP PPTGQSDYTK AWEEYYKKIG QQPQQPGAPP QQDYTKAWEE
651 YYKKQAQVAT GGGPGAPPGS QPDYSAAWAE YYRQQAAYYG QTPGPGGPQP
701 PPTQQGQQQA Q
```

Motifs with high probability
Motifs with low probability
Overlapping Motifs

No.	Pos.	Group	Score	No.	Pos.	Group	Score
1	K359	GVRIQ **FKQD** DGTGP	0.85	5	K251	AGLVI **GKGG** ETIKQ	0.5
2	K87	ARQIA **AKIG** GDAAT	0.62	6	K244	IMIPA **GKAG** LVIGK	0.5
3	K494	KQLIE **EKIE** GPLCP	0.5	7	K435	FSIPT **HKCG** LVIGR	0.34
4	K473	NGDPN **FKLF** IIRGS	0.5	8	K628	WEEYY **KKIG** QQPQQ	0.31
