# Supplementary material for: SUMO1 modification of KHSRP regulates tumorigenesis by preventing the TL-G-Rich miRNA biogenesis
Source: Mol Cancer. 2017 Oct 11;16:157. doi: 10.1186/s12943-017-0724-6 (PMC5637259; doi:10.1186/s12943-017-0724-6)
Supplement: Supplementary file 6 — Table S1. The transcript expressions extracted from TCGA database is presented in the normalized FPKM (Fragments Per Kilobase of transcript per Milllion fragments mapped) (PDF 92 kb) [file 12943_2017_724_MOESM6_ESM.pdf]

Additional file 6: Table S1. The transcripts expression extracted from TCGA databank was presented in the normalized FPKM (Fragments Per Kilobase of transcript per Million fragments mapped)			
id	KHSRP	UBC9	gleason score
TCGA-QU-A6IO-01A-11R-A31N-07	3432.11223	6350.93846	6
TCGA-G9-6347-01A-11R-A31N-07	3841.6812	7888.5824	6
TCGA-J4-A67O-01A-11R-A30B-07	4617.95725	6751.87243	7
TCGA-QU-A6IL-01A-11R-A31N-07	4735.78649	6620.0982	7
TCGA-QU-A6IM-01A-11R-A31N-07	5048.61868	6947.54804	7
TCGA-HC-A632-01A-11R-A29R-07	5153.10876	7079.38216	9
TCGA-G9-6379-01A-11R-A31N-07	5293.52886	6563.65823	7
TCGA-J4-A67N-01A-11R-A30B-07	5337.38397	6399.89439	7
TCGA-J4-A67Q-01A-21R-A30B-07	5424.47484	7272.58819	6
TCGA-J4-A67L-01A-11R-A30B-07	5514.01999	5983.71617	7
TCGA-HC-A48F-01A-11R-A250-07	5548.79198	6744.32232	8
TCGA-EJ-A65M-01A-11R-A29R-07	5598.36483	5377.0082	6
TCGA-EJ-5525-01A-01R-1580-07	5612.37297	5450.96682	9
TCGA-KK-A7AW-01A-11R-A32O-07	5647.91535	6842.28606	7
TCGA-J4-A67M-01A-11R-A30B-07	5720.08705	6180.34761	7
TCGA-HC-A6AP-01A-11R-A30B-07	5761.88048	5881.20542	7
TCGA-EJ-A46F-01A-31R-A250-07	5855.05817	6539.54227	8
TCGA-G9-6498-01A-12R-A311-07	5865.79361	6520.01969	7
TCGA-ZG-A9L9-01A-11R-A41O-07	5915.42711	7546.15749	9
TCGA-QU-A6IN-01A-11R-A31N-07	5938.45157	8105.92229	7
TCGA-KK-A6E4-01A-11R-A30B-07	5976.0856	7372.15567	7
TCGA-HC-A6AN-01A-11R-A30B-07	5983.83513	5877.80217	7
TCGA-M7-A723-01A-12R-A32O-07	6115.44487	5795.24719	7
TCGA-HC-A6AS-01A-11R-A30B-07	6155.05144	6277.75333	7
TCGA-HC-A6AQ-01A-11R-A30B-07	6168.68801	6281.7824	7
TCGA-EJ-7312-01B-21R-A32O-07	6169.72206	5635.40851	7
TCGA-EJ-A65B-01A-12R-A30B-07	6172.25805	5778.43237	9
TCGA-J4-A67K-01A-21R-A30B-07	6189.15844	6743.6419	7
TCGA-J4-A67R-01A-21R-A30B-07	6202.67567	6513.46197	7
TCGA-KK-A7B0-01A-11R-A32O-07	6215.93121	6764.03935	9
TCGA-KK-A7AY-01A-11R-A33R-07	6231.64126	6317.93491	7
TCGA-KK-A6E7-01A-11R-A31N-07	6236.82755	4865.57905	9
TCGA-KK-A59V-01A-11R-A29R-07	6278.44661	6726.98992	9
TCGA-EJ-7325-01B-11R-A32O-07	6306.18199	7831.0474	7
TCGA-FC-A66V-01A-21R-A30B-07	6315.34668	5914.49969	7
TCGA-FC-A6HD-01A-11R-A31N-07	6322.34873	5166.80955	7
TCGA-V1-A8MF-01A-11R-A36G-07	6329.01727	5103.70405	6
TCGA-MG-AAMC-01A-11R-A41O-07	6353.62061	6055.81929	9
TCGA-M7-A722-01A-12R-A36G-07	6422.76786	5996.79931	8
TCGA-KK-A6E5-01A-11R-A311-07	6432.97855	5548.49567	7
TCGA-EJ-5521-01A-01R-1580-07	6435.93147	6110.95144	7
TCGA-VP-A872-01A-11R-A352-07	6483.51393	7369.68019	8
TCGA-G9-7521-01A-11R-2263-07	6488.57542	5336.63673	8
TCGA-H9-A6BY-01A-11R-A30B-07	6543.41379	6632.29047	7
TCGA-EJ-8472-01A-11R-2403-07	6599.77618	6333.60803	8

TCGA-EJ-5516-01A-01R-1580-07	6640.52677	5041.4027	7
TCGA-G9-6373-01A-11R-1789-07	6643.37833	6877.84616	7
TCGA-X4-A8KQ-01A-12R-A36G-07	6713.46687	5626.77134	9
TCGA-HC-A6AO-01A-11R-A30B-07	6717.3128	5514.24022	7
TCGA-J9-A52B-01A-11R-A26U-07	6895.49149	6399.72816	9
TCGA-HC-A6AL-01A-11R-A30B-07	6897.03716	6001.36176	7
TCGA-EJ-AB20-01A-12R-A41O-07	6946.31808	7383.90947	6
TCGA-J9-A52E-01A-11R-A26U-07	6980.51707	7109.42378	9
TCGA-X4-A8KS-01A-12R-A36G-07	7054.8948	6094.99441	7
TCGA-G9-7525-01A-31R-2263-07	7063.83955	5602.8514	7
TCGA-G9-6354-01A-11R-A311-07	7067.93898	5363.87835	7
TCGA-EJ-8469-01A-11R-2403-07	7093.6185	6801.04251	9
TCGA-M7-A71Z-01A-12R-A32O-07	7106.61968	6638.34557	7
TCGA-J9-A52C-01A-11R-A26U-07	7119.4765	5531.38309	9
TCGA-KK-A8IF-01A-11R-A36G-07	7166.03722	7263.7115	7
TCGA-VP-A875-01A-31R-A352-07	7170.13723	7701.15249	7
TCGA-EJ-5515-01A-01R-1580-07	7187.3599	4480.81014	7
TCGA-ZG-A9LM-01A-11R-A41O-07	7203.35658	5468.59413	9
TCGA-HC-A631-01A-11R-A29R-07	7214.08568	5889.84184	9
TCGA-KK-A7B4-01A-11R-A32O-07	7224.53944	7054.74051	9
TCGA-HC-A6HY-01A-11R-A31N-07	7296.2003	6123.45236	7
TCGA-EJ-A65D-01A-11R-A30B-07	7312.69576	6703.66938	8
TCGA-V1-A9OT-01A-11R-A41O-07	7335.33629	7601.02542	6
TCGA-ZG-A9NI-01A-11R-A41O-07	7375.35373	5954.21833	9
TCGA-EJ-5507-01A-01R-1580-07	7375.69376	4664.82349	9
TCGA-V1-A9Z8-01A-11R-A41O-07	7381.86199	4964.63877	9
TCGA-ZG-A9LU-01A-11R-A41O-07	7429.96122	5757.13555	9
TCGA-FC-A5OB-01A-11R-A29R-07	7432.89919	3013.58214	9
TCGA-HC-A9TE-01A-11R-A41O-07	7461.53401	6145.06752	9
TCGA-HC-7817-01B-11R-A29R-07	7473.73626	5982.47506	7
TCGA-ZG-A8QZ-01A-11R-A37L-07	7515.46017	6148.28082	9
TCGA-HC-A8CY-01A-11R-A36G-07	7525.89567	7682.73702	9
TCGA-KK-A59X-01A-11R-A29R-07	7544.9387	4801.23551	9
TCGA-XK-AAK1-01A-11R-A41O-07	7595.76734	5210.15596	7
TCGA-EJ-5512-01A-01R-1580-07	7600.19029	5426.00868	7
TCGA-ZG-A9N3-01A-11R-A41O-07	7622.96458	6245.67829	9
TCGA-ZG-A9LS-01A-12R-A41O-07	7661.68993	5761.90796	9
TCGA-G9-7519-01A-11R-2263-07	7670.49265	4675.60061	7
TCGA-J9-A52D-01A-11R-A29R-07	7679.5958	5065.73457	9
TCGA-KK-A6E8-01A-11R-A31N-07	7718.68328	5313.42287	9
TCGA-HC-8262-01A-11R-2263-07	7738.31583	6175.00859	8
TCGA-YL-A8SO-01B-31R-A37L-07	7739.26406	8120.71249	9
TCGA-EJ-A65G-01A-21R-A29R-07	7745.72352	4864.48092	8
TCGA-ZG-A8QX-01A-11R-A37L-07	7750.54907	5433.70819	6
TCGA-FC-7961-01A-11R-A29R-07	7802.85867	5034.56655	9
TCGA-CH-5741-01A-11R-1580-07	7813.83999	4957.6024	9
TCGA-V1-A8WW-01A-11R-A37L-07	7816.6615	5841.25297	9
TCGA-EJ-5542-01A-01R-1580-07	7828.15286	5902.1199	7
TCGA-ZG-A9M4-01A-11R-A41O-07	7831.72339	6916.2973	9
TCGA-HC-8216-01A-11R-A29R-07	7844.04495	5357.78615	7
TCGA-ZG-A9L0-01A-11R-A41O-07	7887.22198	5966.95275	9

TCGA-ZG-A9LZ-01A-11R-A41O-07	7898.24112	6110.9062	9
TCGA-EJ-7318-01B-11R-A32O-07	7899.04751	5995.35393	7
TCGA-EJ-8474-01A-11R-2403-07	7900.10528	5785.67366	8
TCGA-G9-7523-01A-11R-2263-07	7912.1064	5631.52892	10
TCGA-EJ-A8FO-01A-21R-A36G-07	7924.02265	5323.28521	7
TCGA-YL-A8HM-01A-11R-A36G-07	7924.14457	5451.65021	9
TCGA-KK-A8I4-01A-11R-A36G-07	7932.4284	7490.74045	7
TCGA-EJ-5499-01A-01R-1580-07	7939.48666	5546.56911	7
TCGA-G9-6366-01A-11R-2118-07	7941.8376	5495.08599	7
TCGA-HC-8213-01A-11R-A29R-07	7944.1548	5212.25435	6
TCGA-CH-5788-01A-11R-1580-07	7947.17033	5986.22551	7
TCGA-KK-A8I7-01A-21R-A36G-07	8001.7524	6124.73444	9
TCGA-G9-6332-01A-11R-1789-07	8014.34446	5010.11419	7
TCGA-EJ-5526-01A-01R-1580-07	8014.57407	4319.96422	8
TCGA-M7-A71Y-01A-22R-A32O-07	8017.38726	6087.99956	7
TCGA-VN-A88L-01A-11R-A352-07	8021.86212	5721.81041	7
TCGA-KK-A5A1-01A-11R-A29R-07	8065.92827	5233.99255	9
TCGA-G9-6339-01A-12R-A311-07	8093.39787	5216.98243	7
TCGA-HC-7232-01A-11R-2118-07	8102.12809	5990.41253	9
TCGA-G9-6362-01A-11R-1789-07	8107.13499	5081.71912	7
TCGA-J9-A8CK-01A-11R-A352-07	8112.78147	6120.26994	9
TCGA-J4-8200-01A-11R-A29R-07	8126.90931	4564.14566	7
TCGA-ZG-A9L6-01A-11R-A41O-07	8132.45996	5270.19147	9
TCGA-EJ-5517-01A-01R-1580-07	8136.00265	4670.85153	6
TCGA-VP-AA1N-01A-31R-A41O-07	8139.42953	4936.59516	9
TCGA-KK-A6E0-01A-11R-A311-07	8158.50449	4007.54076	9
TCGA-G9-6343-01A-21R-1965-07	8168.2134	5351.7233	7
TCGA-ZG-A9MC-01A-31R-A41O-07	8170.84646	6711.65955	9
TCGA-G9-A9S0-01A-11R-A41O-07	8171.96221	5109.28659	8
TCGA-EJ-5504-01A-01R-1580-07	8177.91079	4930.82059	7
TCGA-XJ-A83F-01A-11R-A352-07	8182.65544	5429.97658	7
TCGA-EJ-8468-01A-21R-2403-07	8184.55423	4773.13896	8
TCGA-CH-5737-01A-11R-1580-07	8200.60377	4565.80358	7
TCGA-KK-A8IC-01A-11R-A36G-07	8204.16531	5159.33735	9
TCGA-EJ-A7NF-01A-11R-A33R-07	8206.53215	5980.12766	7
TCGA-XK-AAJT-01A-11R-A41O-07	8207.28989	6214.69507	7
TCGA-2A-A8VL-01A-21R-A37L-07	8225.75353	4798.1579	6
TCGA-EJ-5505-01A-01R-1580-07	8245.92803	4474.68325	7
TCGA-G9-6361-01A-21R-1965-07	8250.48109	6191.41612	7
TCGA-J4-AATV-01A-11R-A41O-07	8270.18867	5965.0159	6
TCGA-XK-AAIR-01A-11R-A41O-07	8282.22802	5043.14208	8
TCGA-G9-6336-01A-11R-1789-07	8284.28787	5126.88207	7
TCGA-KK-A8IB-01A-11R-A36G-07	8296.10164	5758.48306	9
TCGA-ZG-A9L4-01A-11R-A41O-07	8302.50495	5437.26737	9
TCGA-G9-6351-01A-21R-1965-07	8304.43528	4838.75331	7
TCGA-V1-A9OX-01A-11R-A41O-07	8308.18621	4996.25308	8
TCGA-4L-AA1F-01A-11R-A41O-07	8308.76796	5044.07421	8
TCGA-J9-A8CP-01A-11R-A352-07	8316.10057	5332.85102	7
TCGA-TK-A8OK-01A-22R-A36G-07	8316.34865	6270.69091	7
TCGA-EJ-5514-01A-01R-1580-07	8337.30602	4128.63551	9
TCGA-YL-A9WY-01A-11R-A41O-07	8344.45513	5860.70699	9

TCGA-V1-A8MK-01A-11R-A36G-07	8344.95391	4922.31489	6
TCGA-XJ-A9DK-01A-11R-A37L-07	8355.77328	5702.79444	8
TCGA-VN-A88K-01A-11R-A352-07	8356.79495	5465.43352	8
TCGA-KK-A8IA-01A-11R-A36G-07	8388.53335	5735.09736	9
TCGA-YL-A8SI-01A-11R-A41O-07	8390.49171	4818.57818	9
TCGA-TP-A8TT-01A-12R-A41O-07	8391.58068	4725.53403	7
TCGA-KK-A6E6-01A-11R-A311-07	8400.72518	3973.48996	9
TCGA-V1-A9ZI-01A-11R-A41O-07	8416.53129	6195.68748	9
TCGA-ZG-A8QW-01A-11R-A37L-07	8447.4504	5147.55744	9
TCGA-KK-A59Y-01A-11R-A26U-07	8448.46242	5213.2472	9
TCGA-J4-A6G1-01A-11R-A311-07	8453.19819	5940.433	8
TCGA-XJ-A83G-01A-11R-A352-07	8459.75603	4890.46864	7
TCGA-XJ-A9DQ-01A-11R-A37L-07	8469.00952	5314.67193	6
TCGA-ZG-A9L2-01A-31R-A41O-07	8469.25234	5733.42932	9
TCGA-ZG-A9L1-01A-11R-A41O-07	8473.72248	6020.503	9
TCGA-VN-A88I-01A-11R-A352-07	8477.50817	6276.14878	8
TCGA-G9-6499-01A-12R-1965-07	8479.5238	4509.3023	9
TCGA-CH-5743-01A-21R-1580-07	8488.75083	5643.84175	7
TCGA-V1-A9OA-01A-11R-A41O-07	8491.93498	6483.65656	9
TCGA-XK-AAJU-01A-11R-A41O-07	8508.49369	5263.51822	7
TCGA-KK-A7AV-01A-11R-A32O-07	8510.94042	5256.71568	7
TCGA-M7-A720-01A-12R-A32O-07	8524.79264	5276.33062	6
TCGA-EJ-A65E-01A-11R-A29R-07	8524.8837	4208.94982	7
TCGA-EJ-A46E-01A-31R-A250-07	8527.25461	6102.03249	8
TCGA-G9-6338-01A-12R-1965-07	8528.14438	4912.72044	7
TCGA-KK-A8IH-01A-11R-A36G-07	8528.37931	5254.98967	7
TCGA-EJ-5524-01A-01R-1580-07	8529.67144	5129.88998	9
TCGA-V1-A9O7-01A-21R-A41O-07	8534.57837	6070.47077	9
TCGA-G9-6385-01A-11R-1789-07	8536.27238	4801.90564	7
TCGA-HC-A8D0-01A-11R-A36G-07	8541.39611	5922.41415	7
TCGA-CH-5789-01A-11R-1580-07	8542.98134	4816.27648	7
TCGA-G9-6329-01A-13R-1965-07	8545.15725	5503.0235	7
TCGA-ZG-A8QY-01A-11R-A37L-07	8550.27301	5128.44969	9
TCGA-G9-7522-01A-11R-2263-07	8555.4801	4396.32212	7
TCGA-EJ-A46B-01A-31R-A250-07	8566.35721	4566.37801	8
TCGA-EJ-5522-01A-01R-1580-07	8568.67507	4895.55019	7
TCGA-2A-A8VO-01A-11R-A37L-07	8571.27653	4676.18604	6
TCGA-CH-5772-01A-11R-1580-07	8573.07077	3501.22777	9
TCGA-EJ-A8FP-01A-21R-A36G-07	8579.10006	5225.05531	8
TCGA-CH-5740-01A-11R-1580-07	8580.15733	5191.84701	7
TCGA-EJ-A6RC-01A-11R-A32O-07	8582.9807	5605.69797	7
TCGA-KC-A4BR-01A-32R-A32Y-07	8596.90512	5828.0966	9
TCGA-G9-A9S4-01A-11R-A41O-07	8603.65963	6505.52743	8
TCGA-YL-A8HO-01A-11R-A36G-07	8610.32217	5234.26216	7
TCGA-HC-8264-01B-11R-2403-07	8612.22712	4308.39383	9
TCGA-VN-A88R-01A-11R-A36G-07	8614.94737	4262.47876	8
TCGA-KK-A6E1-01A-11R-A311-07	8617.22154	4562.5813	9
TCGA-2A-A8VV-01A-11R-A37L-07	8620.74779	5464.30299	6
TCGA-EJ-5498-01A-01R-1580-07	8624.62908	5585.23634	7
TCGA-KK-A8IG-01A-11R-A36G-07	8626.26892	4978.43195	7
TCGA-G9-A9S7-01A-11R-A41O-07	8629.92079	4617.22528	8

TCGA-EJ-A7NG-01A-31R-A33R-07	8637.44745	5310.77936	7
TCGA-KC-A7FE-01A-12R-A33R-07	8650.51663	5128.83344	7
TCGA-CH-5790-01A-11R-1580-07	8655.29908	4836.94316	7
TCGA-KK-A8I6-01A-11R-A36G-07	8656.39347	5497.95732	7
TCGA-KC-A7FA-01A-21R-A33R-07	8661.06669	5614.13195	7
TCGA-G9-7510-01A-11R-2263-07	8661.93914	4586.3377	8
TCGA-EJ-A65J-01A-11R-A311-07	8661.98559	4358.38882	9
TCGA-V1-A9Z9-01A-21R-A41O-07	8676.33101	4460.70317	9
TCGA-V1-A8MG-01A-11R-A36G-07	8679.97604	5348.23686	7
TCGA-KC-A7F3-01A-21R-A33R-07	8688.80976	5210.4241	7
TCGA-Y6-A8TL-01A-21R-A37L-07	8701.33905	4514.50585	6
TCGA-EJ-5506-01A-01R-1580-07	8703.54851	4815.14679	8
TCGA-G9-6370-01A-11R-1789-07	8707.04046	5026.81638	7
TCGA-EJ-5496-01A-01R-1580-07	8710.73292	4556.84899	7
TCGA-G9-7509-01A-11R-A41O-07	8713.70531	5338.73239	6
TCGA-HC-7747-01A-11R-2118-07	8718.13682	5075.00241	7
TCGA-V1-A9ZR-01A-11R-A41O-07	8721.24058	5551.73846	8
TCGA-G9-6367-01A-11R-1789-07	8723.52748	4591.66713	9
TCGA-YL-A8SQ-01B-11R-A37L-07	8726.75753	4793.54163	9
TCGA-G9-6378-01A-11R-1789-07	8731.83675	4457.12853	7
TCGA-J4-A6M7-01A-11R-A31N-07	8742.51956	5505.47629	7
TCGA-G9-6356-01A-11R-1789-07	8746.06793	5427.72154	9
TCGA-G9-6364-01A-21R-1789-07	8746.64247	5215.13478	7
TCGA-EJ-A7NK-01A-12R-A352-07	8749.69664	5223.81977	7
TCGA-YL-A9WH-01A-11R-A37L-07	8755.16775	6345.76203	9
TCGA-FC-A8O0-01A-41R-A37L-07	8760.48923	5611.90001	6
TCGA-V1-A9OH-01A-11R-A41O-07	8761.43539	5173.86554	8
TCGA-HC-8257-01A-11R-2263-07	8762.00113	4657.88458	7
TCGA-M7-A724-01A-12R-A32O-07	8768.22295	4962.56764	8
TCGA-HI-7171-01A-12R-2118-07	8770.39239	5755.98748	9
TCGA-HI-7168-01A-11R-2118-07	8773.77193	5831.4797	9
TCGA-V1-A8MM-01A-11R-A37L-07	8775.61061	5626.01296	7
TCGA-VP-A878-01A-31R-A352-07	8775.98557	4828.09434	9
TCGA-HC-8261-01A-11R-2263-07	8776.01153	4044.88308	7
TCGA-CH-5765-01A-11R-1580-07	8776.85391	4778.15679	7
TCGA-KC-A7F5-01A-11R-A33R-07	8788.03503	5989.20948	7
TCGA-KK-A7B3-01A-11R-A33R-07	8792.70846	5585.66814	9
TCGA-HC-A8D1-01A-11R-A36G-07	8793.39095	5314.35204	7
TCGA-CH-5762-01A-11R-1580-07	8797.90059	5031.59184	7
TCGA-2A-AAYO-01A-11R-A41O-07	8798.19631	4592.57537	6
TCGA-XA-A8JR-01A-11R-A36G-07	8803.51078	4529.27861	7
TCGA-CH-5791-01A-11R-1580-07	8807.00161	4723.21648	7
TCGA-XK-AAJR-01A-11R-A41O-07	8808.98976	4581.39543	7
TCGA-YL-A8SK-01B-21R-A37L-07	8813.52856	5850.55006	9
TCGA-EJ-A46D-01A-21R-A32Y-07	8820.60427	5006.37991	8
TCGA-ZG-A9ND-01A-11R-A41O-07	8827.37829	4208.84494	9
TCGA-XJ-A9DI-01A-11R-A37L-07	8828.92435	5660.55082	9
TCGA-ZG-A9LY-01A-11R-A41O-07	8833.8177	4858.1341	9
TCGA-EJ-AB27-01A-11R-A41O-07	8836.28014	4970.33407	6
TCGA-CH-5739-01A-11R-1580-07	8836.76468	5836.84011	7
TCGA-VP-A87J-01A-11R-A352-07	8841.21542	5119.69136	7

TCGA-EJ-A7NN-01A-11R-A33R-07	8841.36119	4022.53483	7
TCGA-EJ-A8FU-01A-11R-A36G-07	8858.87528	5716.38161	8
TCGA-YL-A8HL-01A-11R-A36G-07	8865.21158	4684.28764	9
TCGA-G9-6353-01A-11R-1965-07	8872.64438	5122.08752	7
TCGA-YL-A8SH-01B-11R-A37L-07	8873.08971	4900.19579	7
TCGA-CH-5745-01A-11R-1580-07	8873.1084	5076.20251	7
TCGA-2A-A8VX-01A-11R-A37L-07	8875.03486	5112.45687	8
TCGA-KK-A7AZ-01A-12R-A32O-07	8875.16529	5843.45042	7
TCGA-J4-A83N-01A-11R-A352-07	8878.51069	4843.70632	7
TCGA-G9-6369-01A-21R-1965-07	8895.12257	3802.08333	7
TCGA-KK-A8I8-01A-11R-A36G-07	8897.40073	5633.69019	9
TCGA-EJ-A65F-01A-21R-A311-07	8909.39562	4819.52117	9
TCGA-VN-A88M-01A-11R-A352-07	8911.39416	5833.61363	7
TCGA-HC-7213-01A-11R-2118-07	8915.61061	6329.59335	9
TCGA-EJ-A46G-01A-31R-A26U-07	8918.39276	6162.30074	8
TCGA-XQ-A8TA-01A-11R-A36G-07	8927.08959	3673.23186	10
TCGA-EJ-5497-01A-02R-1580-07	8936.33522	4626.5117	7
TCGA-HC-7742-01A-11R-2118-07	8942.25631	5026.27257	7
TCGA-KK-A59Z-01A-12R-A26U-07	8945.29439	4715.15584	7
TCGA-EJ-A7NJ-01A-22R-A352-07	8948.44597	4673.08331	8
TCGA-KK-A8IL-01A-11R-A36G-07	8960.82698	5218.20355	9
TCGA-EJ-A8FS-01A-11R-A352-07	8963.58849	4312.19895	7
TCGA-HC-7079-01A-11R-1965-07	8969.31043	5747.41933	7
TCGA-YL-A8SA-01A-21R-A37L-07	8970.24421	5181.1012	8
TCGA-YL-A8HK-01A-11R-A36G-07	8980.62344	5479.80842	9
TCGA-YL-A8SR-01B-11R-A37L-07	8984.35283	4291.18905	9
TCGA-HC-A76X-01A-11R-A33R-07	8993.92909	5437.68161	7
TCGA-J4-AAU2-01A-11R-A41O-07	8994.51108	4272.11736	6
TCGA-V1-A9OY-01A-11R-A41O-07	9001.96516	5774.81536	7
TCGA-YL-A9WX-01A-21R-A41O-07	9012.24624	5302.4211	9
TCGA-KK-A8IM-01A-11R-A36G-07	9019.14808	5144.64786	7
TCGA-2A-AAYU-01A-11R-A41O-07	9019.62272	5244.12579	6
TCGA-V1-A8WV-01A-11R-A37L-07	9019.90713	6692.60798	9
TCGA-EJ-7218-01B-11R-A32O-07	9028.38651	4953.73377	7
TCGA-V1-A8WL-01A-11R-A37L-07	9032.39158	4493.67846	7
TCGA-G9-6371-01A-11R-1789-07	9070.72802	4670.50247	6
TCGA-V1-A8WN-01A-11R-A37L-07	9071.37726	4783.98944	6
TCGA-J9-A8CM-01A-11R-A352-07	9072.87198	5308.13356	9
TCGA-CH-5750-01A-11R-1580-07	9072.94437	5237.56162	7
TCGA-HC-8265-01A-11R-2263-07	9077.95237	4844.98999	8
TCGA-CH-5746-01A-11R-1580-07	9082.40947	4875.20061	7
TCGA-ZG-A9LB-01A-11R-A41O-07	9085.21021	5146.41538	9
TCGA-J4-8198-01A-11R-2263-07	9085.39793	5436.3159	7
TCGA-V1-A9ZK-01A-11R-A41O-07	9094.76014	4326.90852	8
TCGA-EJ-5495-01A-01R-1580-07	9099.50072	5181.32709	8
TCGA-VP-A87D-01A-11R-A352-07	9100.2684	4976.79827	9
TCGA-EJ-5510-01A-01R-1580-07	9101.72848	5135.3357	7
TCGA-VP-A87C-01A-11R-A352-07	9102.22006	5413.091	7
TCGA-KK-A8IJ-01A-11R-A352-07	9108.49287	4295.51698	7
TCGA-XJ-A83H-01A-11R-A352-07	9121.57846	5039.9562	7
TCGA-KK-A8II-01A-11R-A36G-07	9129.47645	4791.63903	9

TCGA-EJ-5508-01A-02R-1580-07	9135.72896	4493.37443	7
TCGA-J4-A6G3-01A-11R-A311-07	9136.14259	4702.56385	8
TCGA-H9-7775-01A-11R-2118-07	9142.32914	4442.36254	7
TCGA-HC-7750-01A-11R-2118-07	9145.20487	4991.84803	7
TCGA-VP-A876-01A-11R-A352-07	9158.57076	5007.56567	8
TCGA-XJ-A9DX-01A-11R-A37L-07	9165.1772	5149.02335	9
TCGA-ZG-A9L5-01A-12R-A41O-07	9174.21569	6274.89825	9
TCGA-YL-A8SB-01A-31R-A37L-07	9174.92389	4609.64109	9
TCGA-G9-6494-01A-11R-1789-07	9175.30062	4370.27049	7
TCGA-KK-A7B1-01A-11R-A32O-07	9176.90401	5097.09126	7
TCGA-HC-7821-01A-12R-2118-07	9177.18377	4939.82442	8
TCGA-YL-A8S9-01A-11R-A37L-07	9177.3546	4787.29033	9
TCGA-V1-A8ML-01A-11R-A37L-07	9180.08415	4645.90932	7
TCGA-CH-5744-01A-11R-1580-07	9185.53376	5141.15968	7
TCGA-KK-A7AU-01A-11R-A32O-07	9213.85948	4902.60329	9
TCGA-YL-A8SL-01B-21R-A37L-07	9220.7307	5802.72383	8
TCGA-KK-A8IK-01A-11R-A36G-07	9223.4436	5388.16809	9
TCGA-HC-7231-01A-11R-2118-07	9228.17627	4632.81592	7
TCGA-V1-A9Z7-01A-11R-A41O-07	9230.09343	4668.46179	9
TCGA-M7-A725-01A-12R-A32O-07	9235.42169	4478.22155	7
TCGA-EJ-7791-01A-11R-2118-07	9238.05747	4767.23777	7
TCGA-M7-A721-01A-12R-A32O-07	9239.42932	4839.11323	7
TCGA-V1-A8X3-01A-11R-A37L-07	9254.84003	4773.00147	7
TCGA-CH-5738-01A-11R-1580-07	9255.0891	4754.30404	6
TCGA-KK-A8I9-01A-11R-A36G-07	9262.21339	4712.50971	8
TCGA-2A-AAYF-01A-11R-A41O-07	9263.12409	3489.27545	7
TCGA-CH-5764-01A-21R-1580-07	9265.60066	4625.35221	7
TCGA-YL-A8HJ-01A-11R-A36G-07	9281.67845	5904.40057	9
TCGA-EJ-A8FN-01A-11R-A352-07	9282.12023	4756.45724	7
TCGA-J4-A83L-01A-11R-A352-07	9292.26064	4460.96545	7
TCGA-XK-AAJA-01A-11R-A41O-07	9294.07759	5050.64592	7
TCGA-KK-A8ID-01A-11R-A36G-07	9294.22087	6318.96665	9
TCGA-XK-AAJ3-01A-11R-A41O-07	9297.65149	4526.15834	8
TCGA-YL-A9WJ-01A-11R-A37L-07	9305.24702	4553.68367	8
TCGA-YL-A8SC-01A-11R-A37L-07	9311.43657	5149.43505	9
TCGA-FC-7708-01A-11R-2118-07	9315.4644	4437.58525	7
TCGA-CH-5771-01A-21R-1580-07	9319.61213	5428.38292	7
TCGA-V1-A9O9-01A-11R-A41O-07	9321.88621	4966.24941	8
TCGA-EJ-A7NH-01A-12R-A33R-07	9328.32068	4716.09894	7
TCGA-HC-7737-01A-11R-2118-07	9328.34147	4663.71392	7
TCGA-KK-A7AP-01A-12R-A33R-07	9333.91679	6828.15313	9
TCGA-2A-A8W1-01A-11R-A37L-07	9345.6172	4461.09038	7
TCGA-HC-A9TH-01A-11R-A41O-07	9356.23692	4866.58658	9
TCGA-V1-A9O5-01A-11R-A41O-07	9358.50035	4877.0168	9
TCGA-J4-A83M-01A-11R-A352-07	9364.55339	5606.69904	7
TCGA-HC-8256-01A-11R-2263-07	9368.76899	3638.19099	7
TCGA-SU-A7E7-01A-22R-A33R-07	9375.35794	4520.80412	8
TCGA-VN-A88N-01A-11R-A36G-07	9375.49802	4225.33824	7
TCGA-KC-A4BV-01A-31R-A26U-07	9433.72931	5348.92443	9
TCGA-J4-A83K-01A-11R-A352-07	9440.5257	5028.81107	6
TCGA-EJ-7786-01A-11R-2118-07	9463.9795	4412.79186	7

TCGA-ZG-A9KY-01A-11R-A41O-07	9483.6521	4114.06543	9
TCGA-CH-5753-01A-11R-1580-07	9486.80579	4404.30219	9
TCGA-XK-AAIV-01A-11R-A41O-07	9492.82269	5548.59032	10
TCGA-KC-A4BL-01A-31R-A250-07	9501.51509	4992.35736	7
TCGA-VP-A87K-01A-11R-A352-07	9502.90739	5103.52139	8
TCGA-G9-6333-01A-12R-1965-07	9507.61473	3946.39611	7
TCGA-XQ-A8TB-01A-11R-A36G-07	9521.53769	5221.69099	9
TCGA-YL-A9WI-01A-11R-A37L-07	9522.14646	5279.26684	9
TCGA-CH-5766-01A-11R-1580-07	9524.97148	4249.02045	7
TCGA-J4-A83I-01A-11R-A36G-07	9534.56932	4771.0109	7
TCGA-TP-A8TV-01A-11R-A41O-07	9542.72516	4027.41389	7
TCGA-EJ-7314-01A-31R-2118-07	9557.66008	4982.39637	7
TCGA-HC-7081-01A-11R-1965-07	9575.03119	5281.76178	9
TCGA-VN-A88P-01A-11R-A352-07	9575.5829	4528.46073	7
TCGA-VP-A879-01A-11R-A352-07	9576.27939	4432.65211	9
TCGA-EJ-A46I-01A-12R-A26U-07	9583.37937	4910.36604	7
TCGA-HC-7820-01A-11R-2118-07	9589.61609	4801.11684	7
TCGA-HC-7210-01A-11R-2118-07	9594.79414	4554.15242	7
TCGA-EJ-5503-01A-01R-1580-07	9609.49608	4831.43067	8
TCGA-EJ-7785-01A-11R-2118-07	9623.82124	5193.79044	7
TCGA-YL-A9WK-01A-11R-A37L-07	9626.24565	4821.81111	9
TCGA-YL-A8S8-01A-11R-A37L-07	9638.03674	4755.47491	9
TCGA-EJ-5511-01A-01R-1580-07	9647.5775	5023.7147	7
TCGA-HC-7745-01A-11R-2118-07	9662.12331	4729.18169	7
TCGA-HC-A76W-01A-11R-A33R-07	9665.1269	5269.63942	7
TCGA-VN-A943-01A-11R-A41O-07	9677.13585	3631.19157	8
TCGA-EJ-7788-01A-11R-2118-07	9687.70055	4641.40247	7
TCGA-YL-A8SP-01B-11R-A37L-07	9689.38374	5693.3823	9
TCGA-KC-A4BN-01A-61R-A250-07	9700.84247	4022.5046	7
TCGA-EJ-5532-01A-01R-1580-07	9712.43031	3712.3388	7
TCGA-Y6-A9XI-01A-11R-A41O-07	9714.66542	3557.14418	8
TCGA-G9-6377-01A-11R-1965-07	9729.62666	4544.05673	7
TCGA-V1-A9OL-01A-11R-A41O-07	9733.1529	4322.99447	9
TCGA-HC-7748-01A-11R-2118-07	9753.71875	4787.97969	6
TCGA-XK-AAJP-01A-11R-A41O-07	9760.53693	3839.23507	7
TCGA-CH-5752-01A-11R-1580-07	9763.95277	4671.48027	8
TCGA-EJ-7781-01A-11R-2118-07	9765.4154	4670.64956	7
TCGA-VP-A87H-01A-11R-A352-07	9766.08937	4897.14199	9
TCGA-VN-A88O-01A-11R-A352-07	9780.7894	3906.88934	7
TCGA-HC-7744-01A-11R-2118-07	9810.12033	4945.96731	7
TCGA-EJ-5494-01A-01R-1580-07	9817.66969	4443.78	7
TCGA-EJ-7115-01A-11R-2118-07	9821.35112	4274.32763	7
TCGA-EJ-7782-01A-11R-2118-07	9832.51356	4259.93425	8
TCGA-HC-A4ZV-01A-11R-A26U-07	9842.60336	3341.58027	9
TCGA-CH-5794-01A-11R-1580-07	9848.15629	4845.54321	7
TCGA-HC-7080-01A-11R-1965-07	9849.63441	3855.14966	7
TCGA-EJ-5530-01A-01R-1580-07	9855.32881	4795.08545	7
TCGA-EJ-5501-01A-01R-1580-07	9868.12956	4760.48003	7
TCGA-EJ-A46H-01A-31R-A26U-07	9878.42385	5040.01917	7
TCGA-HC-7818-01A-11R-2118-07	9892.72932	4289.8088	7
TCGA-J9-A8CL-01A-11R-A352-07	9894.72876	4057.04894	9

TCGA-CH-5754-01A-11R-1580-07	9934.44437	3970.61193	9
TCGA-HC-7075-01A-11R-1965-07	9940.20102	4101.58974	6
TCGA-HC-7230-01A-11R-2118-07	9942.38907	4250.08474	7
TCGA-EJ-7789-01A-11R-2118-07	9943.80938	4487.66938	7
TCGA-VP-A87B-01A-11R-A352-07	9943.90897	5477.44131	8
TCGA-2A-A8VT-01A-11R-A37L-07	9979.11956	4754.70257	9
TCGA-V1-A8WS-01A-11R-A37L-07	9995.2176	5200.1357	6
TCGA-EJ-5518-01A-01R-1580-07	9995.75752	3947.59239	9
TCGA-HC-7749-01A-11R-2118-07	10011.6923	4239.11165	7
TCGA-J4-AATZ-01A-11R-A41O-07	10020.8894	3946.34275	9
TCGA-FC-A4JI-01A-11R-A250-07	10041.2075	4623.90014	8
TCGA-CH-5761-01A-11R-1580-07	10046.5204	4066.65165	9
TCGA-HC-7209-01A-11R-2118-07	10052.0942	3964.25926	6
TCGA-CH-5751-01A-11R-1580-07	10052.6408	4403.32277	10
TCGA-EJ-7794-01A-11R-2118-07	10062.6667	4414.05543	7
TCGA-YL-A8SJ-01B-11R-A37L-07	10089.5333	4831.32989	9
TCGA-EJ-7123-01A-11R-1965-07	10092.4643	4196.24547	7
TCGA-HC-7233-01A-11R-2118-07	10102.4984	4227.81711	7
TCGA-CH-5792-01A-11R-1580-07	10177.3837	4700.83581	9
TCGA-EJ-5502-01A-01R-1580-07	10207.3465	4498.53764	7
TCGA-HC-7736-01A-11R-2118-07	10244.6897	4165.94502	7
TCGA-EJ-7330-01A-11R-2118-07	10248.2313	4291.91757	7
TCGA-EJ-5509-01A-01R-1580-07	10250.2753	4182.43298	7
TCGA-HC-7212-01A-11R-2118-07	10291.3617	4234.97083	7
TCGA-V1-A9ZG-01A-11R-A41O-07	10363.7682	4907.85799	9
TCGA-KC-A7FD-01A-11R-A33R-07	10398.0363	4395.94589	7
TCGA-EJ-7327-01A-11R-2118-07	10418.9644	4469.46814	7
TCGA-HC-7077-01A-11R-1965-07	10455.7171	4037.52537	6
TCGA-EJ-5519-01A-01R-1580-07	10542.6469	4322.49788	8
TCGA-WW-A8ZI-01A-11R-A37L-07	10643.4556	4340.38268	8
TCGA-YL-A9WL-01A-11R-A41O-07	10829.2378	3912.73406	9
TCGA-XK-AAIW-01A-11R-A41O-07	11173.3766	3760.48558	9
TCGA-YJ-A8SW-01A-11R-A37L-07	11321.8783	2470.16456	9

Transcripts median value: KHSRP is 8732 FPKM and Ubc9 is 5112 FPKM. The value higher than the median value is in red and the value lower than the median value is in green.
