# Supplementary material for: SUMO1 modification of KHSRP regulates tumorigenesis by preventing the TL-G-Rich miRNA biogenesis
Source: Mol Cancer. 2017 Oct 11;16:157. doi: 10.1186/s12943-017-0724-6 (PMC5637259; doi:10.1186/s12943-017-0724-6)
Supplement: Supplementary file 8 — Table S2. MiRNAs expression in DU145 shRNA Ctrl and shKHSRP stabel cell lines (PDF 74 kb) [file 12943_2017_724_MOESM8_ESM.pdf]

Additional file 8: Table S2. The miRNA expression in DU145 shRNA-Control and shRNA-KHSRP stabel cell lines was presented in the normalized RPM (Reads per Million)			
miRNA	precursor	shCtrl (RPM)	shKHSRP (RPM)
hsa-miR-21-5p	hsa-mir-21	324804.44	342541.17
hsa-let-7i-5p	hsa-let-7i	66743.11	49900.27
hsa-let-7f-5p	hsa-let-7f-2	49544.25	47289.82
hsa-let-7f-5p	hsa-let-7f-1	48965.41	46524.02
hsa-miR-30a-5p	hsa-mir-30a	32123.06	31488.56
hsa-miR-7-5p	hsa-mir-7-1	25654.95	37290.64
hsa-miR-7-5p	hsa-mir-7-2	25442.43	36948.44
hsa-miR-7-5p	hsa-mir-7-3	25440.65	36948.35
hsa-let-7g-5p	hsa-let-7g	24701.22	21522.03
hsa-let-7a-5p	hsa-let-7a-3	18978.32	15824.84
hsa-let-7a-5p	hsa-let-7a-1	18877.54	15787.97
hsa-let-7a-5p	hsa-let-7a-2	18870.93	15783.12
hsa-miR-148a-3p	hsa-mir-148a	18727.74	13999.06
hsa-miR-151a-3p	hsa-mir-151a	16250.06	17278.49
hsa-miR-92a-3p	hsa-mir-92a-1	12917.92	14312.29
hsa-miR-92a-3p	hsa-mir-92a-2	12261.01	13469.62
hsa-miR-30d-5p	hsa-mir-30d	10406.05	10579.55
hsa-miR-146a-5p	hsa-mir-146a	9669.05	783.08
hsa-miR-26a-5p	hsa-mir-26a-2	7795.53	8699.49
hsa-miR-26a-5p	hsa-mir-26a-1	7789.18	8691.96
hsa-miR-24-3p	hsa-mir-24-1	7137.15	6087.59
hsa-miR-24-3p	hsa-mir-24-2	7119.61	6061.88
hsa-miR-103a-3p	hsa-mir-103a-2	6756.26	6642.66
hsa-miR-103a-3p	hsa-mir-103a-1	6680.98	6564.21
hsa-miR-200b-3p	hsa-mir-200b	6133.37	3721.53
hsa-miR-30c-5p	hsa-mir-30c-2	5956.56	6476.1
hsa-miR-30c-5p	hsa-mir-30c-1	5952.52	6469.34
hsa-miR-27b-3p	hsa-mir-27b	5942.88	5221.07
hsa-miR-27a-3p	hsa-mir-27a	5556.35	5374.49
hsa-miR-99b-5p	hsa-mir-99b	5333.93	3796.95
hsa-miR-10a-5p	hsa-mir-10a	5278.55	5130.1
hsa-miR-30a-3p	hsa-mir-30a	5071.4	4838.01
hsa-miR-29a-3p	hsa-mir-29a	4573	4841.64
hsa-miR-378a-3p	hsa-mir-378a	4430.92	4452.32
hsa-miR-423-3p	hsa-mir-423	4416.36	5178.49
hsa-miR-148b-3p	hsa-mir-148b	4276.5	2527.68
hsa-miR-222-3p	hsa-mir-222	3730.62	4148.9
hsa-miR-221-3p	hsa-mir-221	3727.02	3349.4
hsa-let-7b-5p	hsa-let-7b	3690.51	2787.94
hsa-miR-23a-3p	hsa-mir-23a	3584.54	3053.14
hsa-miR-182-5p	hsa-mir-182	3407.6	2965.39
hsa-miR-191-5p	hsa-mir-191	3266.81	3303.33
hsa-miR-128-3p	hsa-mir-128-1	3125.71	2355.86
hsa-miR-532-5p	hsa-mir-532	3088.45	3191.54
hsa-miR-26b-5p	hsa-mir-26b	2996.24	2486.64
hsa-miR-98-5p	hsa-mir-98	2865.31	2072.93
hsa-miR-10b-5p	hsa-mir-10b	2825.96	3888.51

hsa-miR-25-3p	hsa-mir-25	2747.8	3185.87
hsa-let-7d-5p	hsa-let-7d	2625.13	2109.62
hsa-miR-128-3p	hsa-mir-128-2	2587.24	1819.88
hsa-miR-183-5p	hsa-mir-183	2481.41	1564.25
hsa-miR-941	hsa-mir-941-1	2464.09	3093.59
hsa-miR-941	hsa-mir-941-2	2464.09	3093.59
hsa-miR-941	hsa-mir-941-3	2464.09	3093.59
hsa-miR-941	hsa-mir-941-4	2464.09	3093.59
hsa-miR-941	hsa-mir-941-5	2464.09	3093.59
hsa-miR-320a	hsa-mir-320a	2302.64	2545.01
hsa-miR-9-5p	hsa-mir-9-3	2166.34	2092.34
hsa-miR-9-5p	hsa-mir-9-2	2165.59	2092.07
hsa-miR-9-5p	hsa-mir-9-1	2165.5	2091.93
hsa-miR-1307-3p	hsa-mir-1307	2143.29	2078.92
hsa-miR-20a-5p	hsa-mir-20a	1829.07	2579.97
hsa-let-7e-5p	hsa-let-7e	1563.7	1185.09
hsa-miR-340-5p	hsa-mir-340	1494.64	1112.4
hsa-miR-17-5p	hsa-mir-17	1493.3	1918.65
hsa-miR-27a-5p	hsa-mir-27a	1465.23	1769.14
hsa-miR-200a-3p	hsa-mir-200a	1339.1	793.92
hsa-miR-30e-5p	hsa-mir-30e	1320.27	1521.26
hsa-miR-22-3p	hsa-mir-22	1316.49	1397.69
hsa-miR-125a-5p	hsa-mir-125a	1298.86	1497.18
hsa-miR-30e-3p	hsa-mir-30e	1065.25	1192.8
hsa-miR-378i	hsa-mir-378i	1037.67	674.2
hsa-miR-101-3p	hsa-mir-101-2	963.94	881.21
hsa-miR-744-5p	hsa-mir-744	952.13	997.31
hsa-miR-106b-3p	hsa-mir-106b	950.22	854.59
hsa-miR-101-3p	hsa-mir-101-1	932.14	848.47
hsa-miR-423-5p	hsa-mir-423	919.22	1059.25
hsa-miR-140-3p	hsa-mir-140	906.96	1019.75
hsa-miR-92b-3p	hsa-mir-92b	905.05	801.81
hsa-let-7c-5p	hsa-let-7c	893.95	585.68
hsa-miR-93-5p	hsa-mir-93	863.04	954.77
hsa-miR-31-5p	hsa-mir-31	778.52	615.2
hsa-miR-1303	hsa-mir-1303	749.34	798.54
hsa-miR-186-5p	hsa-mir-186	748.27	971.28
hsa-miR-192-5p	hsa-mir-192	736.37	864.75
hsa-miR-21-3p	hsa-mir-21	722.82	516.29
hsa-miR-484	hsa-mir-484	717.58	719.73
hsa-miR-99b-3p	hsa-mir-99b	712.3	484.19
hsa-miR-4521	hsa-mir-4521	711.37	1136.89
hsa-miR-429	hsa-mir-429	708.17	296.99
hsa-miR-361-3p	hsa-mir-361	702.08	750.56
hsa-miR-374b-5p	hsa-mir-374b	628.67	488.99
hsa-miR-200a-5p	hsa-mir-200a	621.83	429.5
hsa-let-7a-3p	hsa-let-7a-1	613.39	728.12
hsa-let-7a-3p	hsa-let-7a-3	610.37	726.53
hsa-miR-181b-5p	hsa-mir-181b-2	608.77	483.64
hsa-miR-28-3p	hsa-mir-28	590.74	747.25
hsa-miR-181b-5p	hsa-mir-181b-1	578.08	459.02

hsa-miR-2682-5p	hsa-mir-2682	559.07	338.8
hsa-miR-181a-5p	hsa-mir-181a-1	556.19	547.72
hsa-miR-181a-5p	hsa-mir-181a-2	555.43	547.31
hsa-miR-126-3p	hsa-mir-126	510.62	256.81
hsa-miR-130b-5p	hsa-mir-130b	500.45	395.44
hsa-miR-23b-3p	hsa-mir-23b	469.31	506.41
hsa-miR-185-5p	hsa-mir-185	459.32	454.48
hsa-miR-425-5p	hsa-mir-425	451.24	445.23
hsa-miR-221-5p	hsa-mir-221	448.48	548.13
hsa-miR-769-5p	hsa-mir-769	445.15	329.19
hsa-miR-146b-5p	hsa-mir-146b	428.19	216.95
hsa-miR-3615	hsa-mir-3615	421.75	456.89
hsa-let-7d-3p	hsa-let-7d	419.48	369.41
hsa-miR-30c-2-3p	hsa-mir-30c-2	402.78	367.46
hsa-miR-196b-5p	hsa-mir-196b	377.51	422.65
hsa-miR-32-5p	hsa-mir-32	375.07	266.92
hsa-miR-7974	hsa-mir-7974	372.14	577.33
hsa-miR-16-5p	hsa-mir-16-1	364.1	458.2
hsa-miR-16-5p	hsa-mir-16-2	363.83	457.25
hsa-miR-24-2-5p	hsa-mir-24-2	363.08	334.08
hsa-miR-96-5p	hsa-mir-96	354.19	342.43
hsa-miR-1260b	hsa-mir-1260b	345.05	271.86
hsa-miR-107	hsa-mir-107	343.09	249.78
hsa-miR-16-2-3p	hsa-mir-16-2	332.52	433.62
hsa-miR-29b-3p	hsa-mir-29b-2	322.62	247.74
hsa-miR-29b-3p	hsa-mir-29b-1	322.48	247.69
hsa-miR-30b-5p	hsa-mir-30b	309.12	248.87
hsa-miR-574-5p	hsa-mir-574	301.74	307.6
hsa-miR-197-3p	hsa-mir-197	296.19	238.13
hsa-miR-100-5p	hsa-mir-100	294.59	44.4
hsa-miR-330-3p	hsa-mir-330	292.37	181.89
hsa-miR-1260a	hsa-mir-1260a	285.84	231.78
hsa-miR-196a-5p	hsa-mir-196a-2	285.8	273.91
hsa-miR-363-3p	hsa-mir-363	283.67	432.58
hsa-miR-1180-3p	hsa-mir-1180	271.36	297.99
hsa-miR-99a-5p	hsa-mir-99a	256.62	389.18
hsa-miR-181a-3p	hsa-mir-181a-1	251.96	167.2
hsa-miR-196a-5p	hsa-mir-196a-1	248	234.27
hsa-miR-320b	hsa-mir-320b-1	246.63	230.28
hsa-miR-500a-3p	hsa-mir-500a	244.18	231.14
hsa-miR-374a-5p	hsa-mir-374a	243.47	218.58
hsa-miR-548o-3p	hsa-mir-548o	238.68	394.9
hsa-miR-548o-3p	hsa-mir-548o-2	238.68	394.9
hsa-miR-589-5p	hsa-mir-589	233.17	244.07
hsa-miR-374a-3p	hsa-mir-374a	232.01	190.1
hsa-miR-7706	hsa-mir-7706	224.51	161.76
hsa-miR-15b-5p	hsa-mir-15b	199.1	193.37
hsa-miR-378c	hsa-mir-378c	196.79	162.44
hsa-miR-320b	hsa-mir-320b-2	188.62	184.84
hsa-miR-342-3p	hsa-mir-342	188.31	199.76
hsa-miR-501-3p	hsa-mir-501	181.56	166.29

hsa-miR-140-5p	hsa-mir-140	179.52	285.88
hsa-miR-660-5p	hsa-mir-660	177.92	190.65
hsa-miR-335-3p	hsa-mir-335	167.35	130.69
hsa-miR-32-3p	hsa-mir-32	153.63	132.46
hsa-miR-339-5p	hsa-mir-339	153.14	150.24
hsa-miR-361-5p	hsa-mir-361	151.09	146.93
hsa-miR-331-3p	hsa-mir-331	144.3	166.2
hsa-miR-455-5p	hsa-mir-455	140.83	161.08
hsa-miR-381-3p	hsa-mir-381	137.28	114.01
hsa-miR-148a-5p	hsa-mir-148a	137.24	110.97
hsa-miR-151a-5p	hsa-mir-151a	129.6	119.58
hsa-miR-19b-3p	hsa-mir-19b-2	124.58	194.45
hsa-miR-193a-5p	hsa-mir-193a	123.82	132.83
hsa-miR-19b-3p	hsa-mir-19b-1	121.69	192.14
hsa-miR-181a-2-3p	hsa-mir-181a-2	117.47	130.33
hsa-miR-454-3p	hsa-mir-454	117.12	136.23
hsa-miR-4326	hsa-mir-4326	116.18	143.53
hsa-miR-615-3p	hsa-mir-615	116.1	108.93
hsa-miR-15b-3p	hsa-mir-15b	109.3	97.95
hsa-miR-106b-5p	hsa-mir-106b	108.63	95.28
hsa-miR-345-5p	hsa-mir-345	105.04	107.39
hsa-miR-365a-3p	hsa-mir-365a	94.42	68.2
hsa-miR-365b-3p	hsa-mir-365b	94.42	68.2
hsa-let-7f-1-3p	hsa-let-7f-1	94.29	126.39
hsa-miR-132-3p	hsa-mir-132	92.56	122.03
hsa-miR-340-3p	hsa-mir-340	92.25	84.62
hsa-miR-877-5p	hsa-mir-877	90.25	52.6
hsa-miR-1304-3p	hsa-mir-1304	89.89	89.06
hsa-miR-130a-3p	hsa-mir-130a	89.67	111.19
hsa-miR-301a-5p	hsa-mir-301a	88.74	48.93
hsa-miR-574-3p	hsa-mir-574	87.8	84.76
hsa-miR-502-3p	hsa-mir-502	86.96	86.75
hsa-miR-149-5p	hsa-mir-149	86.65	56.1
hsa-miR-218-5p	hsa-mir-218-1	86.25	164.93
hsa-miR-421	hsa-mir-421	85.36	50.84
hsa-miR-218-5p	hsa-mir-218-2	84.74	160.85
hsa-miR-19a-3p	hsa-mir-19a	80.74	134.19
hsa-miR-548am-3p	hsa-mir-548am	80.7	80.77
hsa-miR-339-3p	hsa-mir-339	78.79	83.62
hsa-miR-362-5p	hsa-mir-362	78.52	99.13
hsa-miR-935	hsa-mir-935	77.28	92.92
hsa-miR-137	hsa-mir-137	75.77	74.64
hsa-miR-330-5p	hsa-mir-330	73.64	44.44
hsa-miR-378d	hsa-mir-378d-2	73.19	62.26
hsa-miR-378d	hsa-mir-378d-1	71.55	60.27
hsa-miR-320c	hsa-mir-320c-1	70.48	57.05
hsa-miR-210-3p	hsa-mir-210	70.31	51.65
hsa-miR-320c	hsa-mir-320c-2	67.51	54.37
hsa-miR-4664-3p	hsa-mir-4664	66.44	54.19
hsa-miR-374b-3p	hsa-mir-374b	66.18	54.74
hsa-miR-194-5p	hsa-mir-194-2	62.93	72.47

hsa-miR-598-3p	hsa-mir-598	62.4	62.45
hsa-miR-22-5p	hsa-mir-22	61.78	73.51
hsa-let-7b-3p	hsa-let-7b	61.25	60.4
hsa-miR-331-5p	hsa-mir-331	60.62	65.03
hsa-miR-31-3p	hsa-mir-31	58.31	46.8
hsa-miR-3613-5p	hsa-mir-3613	58.09	43.22
hsa-miR-10a-3p	hsa-mir-10a	58	48.84
hsa-miR-27b-5p	hsa-mir-27b	57.96	83.62
hsa-miR-28-5p	hsa-mir-28	57.52	62.85
hsa-miR-505-3p	hsa-mir-505	57.16	56.69
hsa-miR-148b-5p	hsa-mir-148b	56.36	45.85
hsa-miR-548k	hsa-mir-548k	55.92	56.55
hsa-miR-34a-5p	hsa-mir-34a	55.25	56.46
hsa-miR-671-3p	hsa-mir-671	54.23	67.52
hsa-miR-425-3p	hsa-mir-425	54.01	40.59
hsa-miR-625-3p	hsa-mir-625	53.96	74.87
hsa-miR-328-3p	hsa-mir-328	53.3	49.48
hsa-miR-18a-5p	hsa-mir-18a	50.85	63.26
hsa-miR-152-3p	hsa-mir-152	50.68	42.22
hsa-miR-1296-5p	hsa-mir-1296	50.54	31.74
hsa-miR-760	hsa-mir-760	50.41	31.47
hsa-miR-200b-5p	hsa-mir-200b	48.59	33.2
hsa-miR-92b-5p	hsa-mir-92b	48.41	41.63
hsa-miR-203a-3p	hsa-mir-203a	47.92	26.35
hsa-miR-181d-5p	hsa-mir-181d	47.12	39.32
hsa-miR-30d-3p	hsa-mir-30d	45.08	54.96
hsa-miR-194-5p	hsa-mir-194-1	44.95	52.2
hsa-miR-548f-3p	hsa-mir-548f-2	44.1	66.53
hsa-miR-548f-3p	hsa-mir-548f-3	44.1	66.53
hsa-miR-652-3p	hsa-mir-652	43.52	30.34
hsa-miR-25-5p	hsa-mir-25	43.35	58.14
hsa-miR-942-5p	hsa-mir-942	43.26	56.96
hsa-miR-106a-5p	hsa-mir-106a	42.95	58.05
hsa-miR-9-3p	hsa-mir-9-1	42.77	43.08
hsa-miR-9-3p	hsa-mir-9-2	42.77	43.08
hsa-miR-9-3p	hsa-mir-9-3	42.33	42.9
hsa-miR-185-3p	hsa-mir-185	41.88	35.37
hsa-miR-1307-5p	hsa-mir-1307	40.59	35.96
hsa-miR-132-5p	hsa-mir-132	39.71	45.67
hsa-miR-409-3p	hsa-mir-409	39.62	22.36
hsa-miR-92a-1-5p	hsa-mir-92a-1	38.33	60.63
hsa-miR-125a-3p	hsa-mir-125a	37.17	34.37
hsa-miR-450b-5p	hsa-mir-450b	37.17	21.22
hsa-miR-424-3p	hsa-mir-424	37.04	48.16
hsa-miR-1278	hsa-mir-1278	36.11	31.34
hsa-miR-576-3p	hsa-mir-576	36.06	35.64
hsa-miR-532-3p	hsa-mir-532	35.93	38
hsa-let-7f-2-3p	hsa-let-7f-2	35.75	41.72
hsa-miR-455-3p	hsa-mir-455	35	48.21
hsa-miR-1271-5p	hsa-mir-1271	34.73	38.95
hsa-miR-195-3p	hsa-mir-195	34.29	40.27

hsa-miR-5100	hsa-mir-5100	34.2	29.25
hsa-miR-1301-3p	hsa-mir-1301	33.75	40.27
hsa-miR-3158-3p	hsa-mir-3158-2	33.67	44.35
hsa-miR-3158-3p	hsa-mir-3158-1	33.62	44.35
hsa-miR-1257	hsa-mir-1257	32.82	61.45
hsa-miR-20b-5p	hsa-mir-20b	32.16	50.7
hsa-miR-130b-3p	hsa-mir-130b	32.16	29.34
hsa-miR-501-5p	hsa-mir-501	31.98	30.88
hsa-miR-1268b	hsa-mir-1268b	31.8	32.38
hsa-miR-629-5p	hsa-mir-629	31.27	13.24
hsa-miR-500a-5p	hsa-mir-500a	30.91	34.19
hsa-miR-379-5p	hsa-mir-379	30.56	18.82
hsa-miR-1268a	hsa-mir-1268a	30.42	31.34
hsa-miR-95-3p	hsa-mir-95	30.29	19.95
hsa-miR-222-5p	hsa-mir-222	29.71	21.77
hsa-miR-365b-5p	hsa-mir-365b	29.67	29.39
hsa-miR-454-5p	hsa-mir-454	29.53	24.03
hsa-let-7e-3p	hsa-let-7e	28.65	26.48
hsa-miR-503-5p	hsa-mir-503	27.76	24.58
hsa-miR-3909	hsa-mir-3909	27.36	29.7
hsa-miR-324-3p	hsa-mir-324	26.78	28.89
hsa-miR-671-5p	hsa-mir-671	26.16	32.61
hsa-miR-30c-1-3p	hsa-mir-30c-1	25.94	29.43
hsa-miR-326	hsa-mir-326	25.58	16.1
hsa-miR-7-1-3p	hsa-mir-7-1	25	32.47
hsa-miR-378a-5p	hsa-mir-378a	25	26.98
hsa-miR-18a-3p	hsa-mir-18a	24.87	36.46
hsa-miR-5701	hsa-mir-5701-1	23.98	21.77
hsa-miR-5701	hsa-mir-5701-2	23.98	21.77
hsa-miR-5701	hsa-mir-5701-3	23.98	21.77
hsa-miR-3074-5p	hsa-mir-3074	22.7	20.27
hsa-miR-378f	hsa-mir-378f	22.07	20.45
hsa-miR-450a-5p	hsa-mir-450a-1	22.03	12.7
hsa-miR-450a-5p	hsa-mir-450a-2	21.98	12.7
hsa-miR-1292-5p	hsa-mir-1292	21.94	33.1
hsa-miR-200c-3p	hsa-mir-200c	21.9	8.48
hsa-miR-128-1-5p	hsa-mir-128-1	21.67	17.73
hsa-miR-5010-3p	hsa-mir-5010	21.54	24.26
hsa-miR-125b-5p	hsa-mir-125b-1	21.1	26.66
hsa-miR-654-3p	hsa-mir-654	20.96	12.7
hsa-miR-1254	hsa-mir-1254-1	20.83	27.39
hsa-miR-125b-5p	hsa-mir-125b-2	20.65	26.48
hsa-miR-1254	hsa-mir-1254-2	20.52	27.21
hsa-miR-324-5p	hsa-mir-324	20.21	15.96
hsa-miR-2277-5p	hsa-mir-2277	19.72	17.6
hsa-miR-449c-5p	hsa-mir-449c	19.63	33.83
hsa-miR-122-5p	hsa-mir-122	19.1	16.1
hsa-miR-4473	hsa-mir-4473	18.79	16.33
hsa-miR-548e-3p	hsa-mir-548e	18.65	17.41
hsa-miR-550a-5p	hsa-mir-550a-1	18.56	19.68
hsa-miR-550a-5p	hsa-mir-550a-2	18.56	19.68

hsa-miR-138-5p	hsa-mir-138-1	18.43	26.89
hsa-miR-550a-3-5p	hsa-mir-550a-1	18.39	19.36
hsa-miR-550a-3-5p	hsa-mir-550a-2	18.39	19.36
hsa-miR-576-5p	hsa-mir-576	18.39	17.69
hsa-miR-301b-3p	hsa-mir-301b	17.81	10.88
hsa-miR-155-5p	hsa-mir-155	17.77	23.81
hsa-miR-215-5p	hsa-mir-215	17.77	15.65
hsa-miR-151b	hsa-mir-151b	17.77	12.2
hsa-miR-2682-3p	hsa-mir-2682	17.41	8.48
hsa-miR-147b	hsa-mir-147b	17.28	18.59
hsa-miR-2110	hsa-mir-2110	17.14	16.82
hsa-miR-188-5p	hsa-mir-188	17.14	16.33
hsa-miR-590-3p	hsa-mir-590	17.1	16.55
hsa-miR-641	hsa-mir-641	16.92	18.46
hsa-miR-543	hsa-mir-543	16.92	8.16
hsa-miR-199b-5p	hsa-mir-199b	16.83	13.88
hsa-miR-3934-5p	hsa-mir-3934	16.7	15.01
hsa-miR-98-3p	hsa-mir-98	16.43	12.56
hsa-miR-93-3p	hsa-mir-93	16.12	20.77
hsa-miR-1908-5p	hsa-mir-1908	16.08	8.89
hsa-miR-7641	hsa-mir-7641-1	15.99	4.99
hsa-miR-7641	hsa-mir-7641-2	15.99	4.99
hsa-miR-486-5p	hsa-mir-486-2	15.59	10.43
hsa-miR-10b-3p	hsa-mir-10b	15.32	19.45
hsa-miR-191-3p	hsa-mir-191	15.19	11.84
hsa-miR-486-5p	hsa-mir-486-1	15.19	9.89
hsa-miR-320d	hsa-mir-320d-1	14.88	17.32
hsa-miR-320d	hsa-mir-320d-2	14.88	17.32
hsa-miR-212-5p	hsa-mir-212	14.83	12.7
hsa-miR-181b-3p	hsa-mir-181b-1	14.66	8.25
hsa-miR-651-5p	hsa-mir-651	14.57	11.75
hsa-miR-889-3p	hsa-mir-889	14.39	6.39
hsa-miR-769-3p	hsa-mir-769	13.77	9.39
hsa-miR-577	hsa-mir-577	13.77	11.02
hsa-miR-3129-3p	hsa-mir-3129	13.55	8.84
hsa-miR-29b-1-5p	hsa-mir-29b-1	13.37	17.46
hsa-miR-4286	hsa-mir-4286	13.37	6.3
hsa-miR-378g	hsa-mir-378g	13.01	13.6
hsa-miR-1246	hsa-mir-1246	12.92	25.49
hsa-miR-7977	hsa-mir-7977	12.66	9.21
hsa-miR-199a-3p	hsa-mir-199a-2	12.48	8.43
hsa-miR-199a-3p	hsa-mir-199a-1	12.48	8.43
hsa-miR-199b-3p	hsa-mir-199b	12.48	8.43
hsa-miR-422a	hsa-mir-422a	12.44	14.1
hsa-miR-15a-5p	hsa-mir-15a	12.3	14.78
hsa-miR-1285-3p	hsa-mir-1285-1	11.95	19.91
hsa-miR-323a-3p	hsa-mir-323a	11.95	6.17
hsa-miR-499a-5p	hsa-mir-499a	11.81	12.56
hsa-miR-548f-3p	hsa-mir-548f-5	11.77	9.75
hsa-miR-548b-5p	hsa-mir-548b	11.59	10.97
hsa-miR-3129-5p	hsa-mir-3129	11.59	6.71

hsa-miR-937-3p	hsa-mir-937	11.5	11.11
hsa-miR-3200-3p	hsa-mir-3200	11.33	6.76
hsa-miR-29c-5p	hsa-mir-29c	11.19	15.55
hsa-miR-663a	hsa-mir-663a	11.1	0.23
hsa-miR-4746-5p	hsa-mir-4746	11.01	12.52
hsa-miR-1285-3p	hsa-mir-1285-2	10.97	18.55
hsa-miR-548f-3p	hsa-mir-548f-1	10.79	7.53
hsa-miR-542-3p	hsa-mir-542	10.7	6.67
hsa-miR-548u	hsa-mir-548u	10.57	8.34
hsa-miR-143-3p	hsa-mir-143	10.53	13.15
hsa-miR-3928-3p	hsa-mir-3928	10.44	9.57
hsa-miR-4741	hsa-mir-4741	10.26	4.85
hsa-miR-16-1-3p	hsa-mir-16-1	10.17	11.29
hsa-miR-411-5p	hsa-mir-411	10.17	10.16
hsa-miR-195-5p	hsa-mir-195	10.13	8.43
