# Supplementary material for: SUMO1 modification of KHSRP regulates tumorigenesis by preventing the TL-G-Rich miRNA biogenesis
Source: Mol Cancer. 2017 Oct 11;16:157. doi: 10.1186/s12943-017-0724-6 (PMC5637259; doi:10.1186/s12943-017-0724-6)
Supplement: Supplementary file 9 — Table S3. A subset of miRNAs biogenesis was downregulated in DU145 shKHSRP stable cell lines (PDF 49 kb) [file 12943_2017_724_MOESM9_ESM.pdf]

Additional file 9 : Table S3. A subset of miRNAs biogenesis was downregulated in DU145 shRNA-KHSRP stabel cell lines					
miRNA	precursor	shCtrl	shKHSRP	shKHSRP/shCtrl	downregulation ratio (%)=(shCtrl-shKHSRP)/shCtrl
hsa-let-7i-5p	hsa-let-7i	66743.11	49900.27	0.747646761	25.24%
hsa-let-7g-5p	hsa-let-7g	24701.22	21522.03	0.871294211	12.87%
hsa-let-7a-5p	hsa-let-7a-3	18978.32	15824.84	0.833837769	16.62%
hsa-let-7a-5p	hsa-let-7a-1	18877.54	15787.97	0.836336196	16.37%
hsa-let-7a-5p	hsa-let-7a-2	18870.93	15783.12	0.836372134	16.36%
hsa-miR-148a-3p	hsa-mir-148a	18727.74	13999.06	0.74750397	25.25%
hsa-miR-146a-5p	hsa-mir-146a	9669.05	783.08	0.080988308	91.90%
hsa-miR-24-3p	hsa-mir-24-1	7137.15	6087.59	0.852944102	14.71%
hsa-miR-24-3p	hsa-mir-24-2	7119.61	6061.88	0.851434278	14.86%
hsa-miR-200b-3p	hsa-mir-200b	6133.37	3721.53	0.606767568	39.32%
hsa-miR-27b-3p	hsa-mir-27b	5942.88	5221.07	0.878542054	12.15%
hsa-miR-99b-5p	hsa-mir-99b	5333.93	3796.95	0.711848487	28.82%
hsa-miR-148b-3p	hsa-mir-148b	4276.5	2527.68	0.591062785	40.89%
hsa-miR-221-3p	hsa-mir-221	3727.02	3349.4	0.898680447	10.13%
hsa-let-7b-5p	hsa-let-7b	3690.51	2787.94	0.755434886	24.46%
hsa-miR-23a-3p	hsa-mir-23a	3584.54	3053.14	0.851752247	14.82%
hsa-miR-182-5p	hsa-mir-182	3407.6	2965.39	0.870228313	12.98%
hsa-miR-128-3p	hsa-mir-128-1	3125.71	2355.86	0.753703958	24.63%
hsa-miR-26b-5p	hsa-mir-26b	2996.24	2486.64	0.829920167	17.01%
hsa-miR-98-5p	hsa-mir-98	2865.31	2072.93	0.723457497	27.65%
hsa-let-7d-5p	hsa-let-7d	2625.13	2109.62	0.803624963	19.64%
hsa-miR-128-3p	hsa-mir-128-2	2587.24	1819.88	0.703405946	29.66%
hsa-miR-183-5p	hsa-mir-183	2481.41	1564.25	0.630387562	36.96%
hsa-let-7e-5p	hsa-let-7e	1563.7	1185.09	0.757875552	24.21%
hsa-miR-340-5p	hsa-mir-340	1494.64	1112.4	0.744259487	25.57%
hsa-miR-200a-3p	hsa-mir-200a	1339.1	793.92	0.592875812	40.71%
hsa-miR-378i	hsa-mir-378i	1037.67	674.2	0.649724864	35.03%
hsa-miR-106b-3p	hsa-mir-106b	950.22	854.59	0.899360148	10.06%

hsa-miR-92b-3p	hsa-mir-92b	905.05	801.81	0.885928954	11.41%
hsa-let-7c-5p	hsa-let-7c	893.95	585.68	0.655159685	34.48%
hsa-miR-31-5p	hsa-mir-31	778.52	615.2	0.790217335	20.98%
hsa-miR-21-3p	hsa-mir-21	722.82	516.29	0.71427188	28.57%
hsa-miR-99b-3p	hsa-mir-99b	712.3	484.19	0.679755721	32.02%
hsa-miR-429	hsa-mir-429	708.17	296.99	0.419376703	58.06%
hsa-miR-374b-5p	hsa-mir-374b	628.67	488.99	0.777816661	22.22%
hsa-miR-200a-5p	hsa-mir-200a	621.83	429.5	0.690703247	30.93%
hsa-miR-181b-5p	hsa-mir-181b-2	608.77	483.64	0.794454392	20.55%
hsa-miR-181b-5p	hsa-mir-181b-1	578.08	459.02	0.794042347	20.60%
hsa-miR-2682-5p	hsa-mir-2682	559.07	338.8	0.606006403	39.40%
hsa-miR-126-3p	hsa-mir-126	510.62	256.81	0.502937605	49.71%
hsa-miR-130b-5p	hsa-mir-130b	500.45	395.44	0.790168848	20.98%
hsa-miR-769-5p	hsa-mir-769	445.15	329.19	0.739503538	26.05%
hsa-miR-146b-5p	hsa-mir-146b	428.19	216.95	0.506667601	49.33%
hsa-let-7d-3p	hsa-let-7d	419.48	369.41	0.880637933	11.94%
hsa-miR-32-5p	hsa-mir-32	375.07	266.92	0.711653825	28.83%
hsa-miR-1260b	hsa-mir-1260b	345.05	271.86	0.787885814	21.21%
hsa-miR-107	hsa-mir-107	343.09	249.78	0.728030546	27.20%
hsa-miR-29b-3p	hsa-mir-29b-2	322.62	247.74	0.767900316	23.21%
hsa-miR-29b-3p	hsa-mir-29b-1	322.48	247.69	0.768078641	23.19%
hsa-miR-30b-5p	hsa-mir-30b	309.12	248.87	0.805091874	19.49%
hsa-miR-197-3p	hsa-mir-197	296.19	238.13	0.803977177	19.60%
hsa-miR-100-5p	hsa-mir-100	294.59	44.4	0.150717947	84.93%
hsa-miR-330-3p	hsa-mir-330	292.37	181.89	0.622122653	37.79%
hsa-miR-1260a	hsa-mir-1260a	285.84	231.78	0.810873216	18.91%
hsa-miR-181a-3p	hsa-mir-181a-1	251.96	167.2	0.663597396	33.64%
hsa-miR-374a-5p	hsa-mir-374a	243.47	218.58	0.897769746	10.22%
hsa-miR-374a-3p	hsa-mir-374a	232.01	190.1	0.819361234	18.06%
hsa-miR-7706	hsa-mir-7706	224.51	161.76	0.720502428	27.95%
hsa-miR-378c	hsa-mir-378c	196.79	162.44	0.825448448	17.46%
hsa-miR-335-3p	hsa-mir-335	167.35	130.69	0.780938154	21.91%
hsa-miR-32-3p	hsa-mir-32	153.63	132.46	0.862201393	13.78%
hsa-miR-381-3p	hsa-mir-381	137.28	114.01	0.830492424	16.95%

hsa-miR-148a-5p	hsa-mir-148a	137.24	110.97	0.808583503	19.14%
hsa-miR-15b-3p	hsa-mir-15b	109.3	97.95	0.896157365	10.38%
hsa-miR-106b-5p	hsa-mir-106b	108.63	95.28	0.877105772	12.29%
hsa-miR-365a-3p	hsa-mir-365a	94.42	68.2	0.722304596	27.77%
hsa-miR-365b-3p	hsa-mir-365b	94.42	68.2	0.722304596	27.77%
hsa-miR-877-5p	hsa-mir-877	90.25	52.6	0.582825485	41.72%
hsa-miR-301a-5p	hsa-mir-301a	88.74	48.93	0.551386072	44.86%
hsa-miR-149-5p	hsa-mir-149	86.65	56.1	0.647432198	35.26%
hsa-miR-421	hsa-mir-421	85.36	50.84	0.595595127	40.44%
hsa-miR-330-5p	hsa-mir-330	73.64	44.44	0.603476372	39.65%
hsa-miR-378d	hsa-mir-378d-2	73.19	62.26	0.850662659	14.93%
hsa-miR-378d	hsa-mir-378d-1	71.55	60.27	0.842348008	15.77%
hsa-miR-320c	hsa-mir-320c-1	70.48	57.05	0.809449489	19.06%
hsa-miR-210-3p	hsa-mir-210	70.31	51.65	0.734603897	26.54%
hsa-miR-320c	hsa-mir-320c-2	67.51	54.37	0.805362169	19.46%
hsa-miR-4664-3p	hsa-mir-4664	66.44	54.19	0.815623119	18.44%
hsa-miR-374b-3p	hsa-mir-374b	66.18	54.74	0.827138108	17.29%
hsa-miR-31-3p	hsa-mir-31	58.31	46.8	0.802606757	19.74%
hsa-miR-3613-5p	hsa-mir-3613	58.09	43.22	0.744017903	25.60%
hsa-miR-10a-3p	hsa-mir-10a	58	48.84	0.842068966	15.79%
hsa-miR-148b-5p	hsa-mir-148b	56.36	45.85	0.813520227	18.65%
hsa-miR-425-3p	hsa-mir-425	54.01	40.59	0.751527495	24.85%
hsa-miR-152-3p	hsa-mir-152	50.68	42.22	0.833070245	16.69%
hsa-miR-1296-5p	hsa-mir-1296	50.54	31.74	0.628017412	37.20%
hsa-miR-760	hsa-mir-760	50.41	31.47	0.624280897	37.57%
hsa-miR-200b-5p	hsa-mir-200b	48.59	33.2	0.683268162	31.67%
hsa-miR-92b-5p	hsa-mir-92b	48.41	41.63	0.859946292	14.01%
hsa-miR-203a-3p	hsa-mir-203a	47.92	26.35	0.549874791	45.01%
hsa-miR-181d-5p	hsa-mir-181d	47.12	39.32	0.834465195	16.55%
hsa-miR-652-3p	hsa-mir-652	43.52	30.34	0.697150735	30.28%
hsa-miR-185-3p	hsa-mir-185	41.88	35.37	0.844555874	15.54%
hsa-miR-1307-5p	hsa-mir-1307	40.59	35.96	0.885932496	11.41%
hsa-miR-409-3p	hsa-mir-409	39.62	22.36	0.564361434	43.56%
hsa-miR-450b-5p	hsa-mir-450b	37.17	21.22	0.570890503	42.91%

hsa-miR-1278	hsa-mir-1278	36.11	31.34	0.867903628	13.21%
hsa-miR-5100	hsa-mir-5100	34.2	29.25	0.855263158	14.47%
hsa-miR-629-5p	hsa-mir-629	31.27	13.24	0.423409018	57.66%
hsa-miR-379-5p	hsa-mir-379	30.56	18.82	0.615837696	38.42%
hsa-miR-95-3p	hsa-mir-95	30.29	19.95	0.658633212	34.14%
hsa-miR-222-5p	hsa-mir-222	29.71	21.77	0.732749916	26.73%
hsa-miR-454-5p	hsa-mir-454	29.53	24.03	0.81374873	18.63%
hsa-miR-503-5p	hsa-mir-503	27.76	24.58	0.885446686	11.46%
hsa-miR-326	hsa-mir-326	25.58	16.1	0.629397967	37.06%
hsa-miR-3074-5p	hsa-mir-3074	22.7	20.27	0.892951542	10.70%
hsa-miR-450a-5p	hsa-mir-450a-1	22.03	12.7	0.576486609	42.35%
hsa-miR-450a-5p	hsa-mir-450a-2	21.98	12.7	0.577797998	42.22%
hsa-miR-200c-3p	hsa-mir-200c	21.9	8.48	0.387214612	61.28%
hsa-miR-128-1-5p	hsa-mir-128-1	21.67	17.73	0.818181818	18.18%
hsa-miR-654-3p	hsa-mir-654	20.96	12.7	0.605916031	39.41%
hsa-miR-324-5p	hsa-mir-324	20.21	15.96	0.789708065	21.03%
hsa-miR-2277-5p	hsa-mir-2277	19.72	17.6	0.892494929	10.75%
hsa-miR-122-5p	hsa-mir-122	19.1	16.1	0.842931937	15.71%
hsa-miR-4473	hsa-mir-4473	18.79	16.33	0.869079297	13.09%
hsa-miR-301b-3p	hsa-mir-301b	17.81	10.88	0.610892757	38.91%
hsa-miR-215-5p	hsa-mir-215	17.77	15.65	0.880697805	11.93%
hsa-miR-151b	hsa-mir-151b	17.77	12.2	0.686550366	31.34%
hsa-miR-2682-3p	hsa-mir-2682	17.41	8.48	0.487076393	51.29%
hsa-miR-543	hsa-mir-543	16.92	8.16	0.482269504	51.77%
hsa-miR-199b-5p	hsa-mir-199b	16.83	13.88	0.824717766	17.53%
hsa-miR-3934-5p	hsa-mir-3934	16.7	15.01	0.898802395	10.12%
hsa-miR-98-3p	hsa-mir-98	16.43	12.56	0.764455265	23.55%
hsa-miR-1908-5p	hsa-mir-1908	16.08	8.89	0.552860697	44.71%
hsa-miR-7641	hsa-mir-7641-1	15.99	4.99	0.312070044	68.79%
hsa-miR-7641	hsa-mir-7641-2	15.99	4.99	0.312070044	68.79%
hsa-miR-486-5p	hsa-mir-486-2	15.59	10.43	0.669018602	33.10%
hsa-miR-191-3p	hsa-mir-191	15.19	11.84	0.779460171	22.05%
hsa-miR-486-5p	hsa-mir-486-1	15.19	9.89	0.651086241	34.89%
hsa-miR-212-5p	hsa-mir-212	14.83	12.7	0.856372218	14.36%

hsa-miR-181b-3p	hsa-mir-181b-1	14.66	8.25	0.562755798	43.72%
hsa-miR-651-5p	hsa-mir-651	14.57	11.75	0.806451613	19.35%
hsa-miR-889-3p	hsa-mir-889	14.39	6.39	0.444058374	55.59%
hsa-miR-577	hsa-mir-577	13.77	11.02	0.800290487	19.97%
hsa-miR-769-3p	hsa-mir-769	13.77	9.39	0.681917211	31.81%
hsa-miR-3129-3p	hsa-mir-3129	13.55	8.84	0.652398524	34.76%
hsa-miR-4286	hsa-mir-4286	13.37	6.3	0.471204188	52.88%
hsa-miR-7977	hsa-mir-7977	12.66	9.21	0.727488152	27.25%
hsa-miR-199a-3p	hsa-mir-199a-2	12.48	8.43	0.675480769	32.45%
hsa-miR-199a-3p	hsa-mir-199a-1	12.48	8.43	0.675480769	32.45%
hsa-miR-199b-3p	hsa-mir-199b	12.48	8.43	0.675480769	32.45%
hsa-miR-323a-3p	hsa-mir-323a	11.95	6.17	0.516317992	48.37%
hsa-miR-548f-3p	hsa-mir-548f-5	11.77	9.75	0.82837723	17.16%
hsa-miR-3129-5p	hsa-mir-3129	11.59	6.71	0.578947368	42.11%
hsa-miR-3200-3p	hsa-mir-3200	11.33	6.76	0.596646072	40.34%
hsa-miR-663a	hsa-mir-663a	11.1	0.23	0.020720721	97.93%
hsa-miR-548f-3p	hsa-mir-548f-1	10.79	7.53	0.697868397	30.21%
hsa-miR-542-3p	hsa-mir-542	10.7	6.67	0.623364486	37.66%
hsa-miR-548u	hsa-mir-548u	10.57	8.34	0.789025544	21.10%
hsa-miR-4741	hsa-mir-4741	10.26	4.85	0.472709552	52.73%
hsa-miR-195-5p	hsa-mir-195	10.13	8.43	0.832181639	16.78%
