# Supplementary material for: SUMO1 modification of KHSRP regulates tumorigenesis by preventing the TL-G-Rich miRNA biogenesis
Source: Mol Cancer. 2017 Oct 11;16:157. doi: 10.1186/s12943-017-0724-6 (PMC5637259; doi:10.1186/s12943-017-0724-6)
Supplement: Supplementary file 10 — Table S4. KHSRP K87R promotes a subset of miRNAs biogenesis in DU145 stable cell lines (PDF 76 kb) [file 12943_2017_724_MOESM10_ESM.pdf]

**Additional file 10: Table S4. KHSRP-K87R promoted a subset of miRNAs biogenesis in DU145 stable cell lines**						Gs in the terminal loop									
						GGGGG	GGGG	GGG	GG	G	GUGG	GUG	GGAG	GAG	
miRNA	precursor	KHSRP-WT	KHSRP-K87R	K87R/WT	upregulation ratio(%)= (K87R-WT) / WT										
hsa-let-7i-5p	hsa-let-7i	44505.83	62924.09	1.413839	41.38%			√	√	√		√	√		
hsa-let-7g-5p	hsa-let-7g	21052.52	26778.8	1.272	27.20%			√					√		
hsa-miR-148a-3p	hsa-mir-148a	13017.69	22059.79	1.694601	69.46%					√				√	
hsa-miR-99b-5p	hsa-mir-99b	3903.84	4862.12	1.245471	24.55%			√		√					
hsa-miR-148b-3p	hsa-mir-148b	2459.28	3030.36	1.232214	23.22%				√	√	√				
hsa-miR-182-5p	hsa-mir-182	2511.88	4386.56	1.746325	74.63%				√				√		
hsa-miR-26b-5p	hsa-mir-26b	2674.64	3243.59	1.21272	21.27%					√		√			
hsa-miR-98-5p	hsa-mir-98	1814.85	2557.91	1.409433	40.94%		√	√	√	√				√	
hsa-miR-183-5p	hsa-mir-183	1579.09	2223.77	1.40826	40.83%					√		√			
hsa-let-7e-5p	hsa-let-7e	1064.01	1267.05	1.190825	19.08%				√	√			√		
hsa-miR-21-3p	hsa-mir-21	656.89	858.83	1.307418	30.74%				√	√					
hsa-miR-32-5p	hsa-mir-32	277.61	429.18	1.545982	54.60%				√	√	√				
hsa-miR-107	hsa-mir-107	282.74	321.13	1.135778	13.58%				√	√			√		
hsa-miR-29b-3p	hsa-mir-29b-2	244.97	355.95	1.453035	45.30%					√					*
hsa-miR-29b-3p	hsa-mir-29b-1	244.97	355.77	1.4523	45.23%					√		√			
hsa-miR-30b-5p	hsa-mir-30b	291.17	349.47	1.200227	20.02%				√	√					
hsa-miR-197-3p	hsa-mir-197	256.49	308.92	1.204413	20.44%					√				√	
hsa-miR-330-3p	hsa-mir-330	127.8	197.99	1.549218	54.92%					√					
hsa-miR-7706	hsa-mir-7706	155.76	250.41	1.607666	60.77%				√	√			√	√	
hsa-miR-32-3p	hsa-mir-32	129.9	144.75	1.114319	11.43%					√		√			
hsa-miR-381-3p	hsa-mir-381	89.1	129.85	1.457351	45.74%				√	√					
hsa-miR-365a-3p	hsa-mir-365a	40.69	83.38	2.049152	104.92%										*
hsa-miR-365b-3p	hsa-mir-365b	40.75	83.38	2.046135	104.61%					√					
hsa-miR-877-5p	hsa-mir-877	39.26	76.09	1.938105	93.81%	√		√	√	√		√			

hsa-miR-301a-5p	hsa-mir-301a	48.85	59.56	1.219243	21.92%					√					
hsa-miR-149-5p	hsa-mir-149	57.84	68.02	1.176003	17.60%			√	√	√		√	√		
hsa-miR-421	hsa-mir-421	43.39	54.99	1.267343	26.73%					√					*
hsa-miR-330-5p	hsa-mir-330	41.63	54.64	1.312515	31.25%					√					
hsa-miR-4664-3p	hsa-mir-4664	53.59	83.18	1.552155	55.22%					√				√	
hsa-miR-374b-3p	hsa-mir-374b	65.23	72.68	1.114211	11.42%					√					*
hsa-miR-31-3p	hsa-mir-31	36.5	67.03	1.836438	83.64%			√		√					
hsa-miR-425-3p	hsa-mir-425	47.47	58.8	1.238677	23.87%			√		√				√	
hsa-miR-760	hsa-mir-760	25.03	40.38	1.613264	61.33%					√					*
hsa-miR-92b-5p	hsa-mir-92b	42.62	56.01	1.314172	31.42%					√					
hsa-miR-629-5p	hsa-mir-629	12.41	18.54	1.493956	49.40%				√	√	√		√		
hsa-miR-301b-3p	hsa-mir-301b	7.83	11.14	1.422733	42.27%					√		√		√	
hsa-miR-2682-3p	hsa-mir-2682	8.99	10.64	1.183537	18.35%			√							
hsa-miR-199b-5p	hsa-mir-199b	8.05	16.06	1.995031	99.50%				√	√					
hsa-miR-486-5p	hsa-mir-486-2	10.53	12.86	1.221273	22.13%			√		√				√	
hsa-miR-486-5p	hsa-mir-486-1	10.2	12.01	1.177451	17.75%					√					
hsa-miR-212-5p	hsa-mir-212	10.7	14.64	1.368224	36.82%			√		√					
hsa-miR-651-5p	hsa-mir-651	13.89	20.17	1.452124	45.21%					√					*
hsa-miR-769-3p	hsa-mir-769	9.54	14.08	1.475891	47.59%					√		√		√	
hsa-miR-3129-3p	hsa-mir-3129	8.6	12.36	1.437209	43.72%					√					
hsa-miR-323a-3p	hsa-mir-323a	5.29	5.95	1.124764	12.48%				√	√					
hsa-miR-548f-3p	hsa-mir-548f-5	8.77	10.38	1.18358	18.36%			√		√					
hsa-miR-3129-5p	hsa-mir-3129	7.11	9.13	1.284107	28.41%					√					
hsa-miR-3200-3p	hsa-mir-3200	4.96	9.01	1.816532	81.65%					√		√			
hsa-miR-548f-3p	hsa-mir-548f-1	7.39	8.25	1.116373	11.64%					√					
hsa-miR-542-3p	hsa-mir-542	4.8	5.66	1.179167	17.92%					√		√			
hsa-miR-4741	hsa-mir-4741	5.46	7.46	1.3663	36.63%				√						

Note: * represents a miRNA that harbors only one single G or none G in its precursor terminal loop.
