# Supplementary material for: SUMO1 modification of KHSRP regulates tumorigenesis by preventing the TL-G-Rich miRNA biogenesis
Source: Mol Cancer. 2017 Oct 11;16:157. doi: 10.1186/s12943-017-0724-6 (PMC5637259; doi:10.1186/s12943-017-0724-6)
Supplement: Supplementary file 16 — Table S5. All primers or oligonucleotides used in this study (PDF 245 kb) [file 12943_2017_724_MOESM16_ESM.pdf]

Additional file 16: Table S5. All primers or oligonucleotides used in this study.

1) Primers used for construction of HA-KHSRP and Flag-KHSRP

hKSRP	Primer	sequences (5’-3’)
HA-KHSRP aa 68-712	Forward	ccggaattcggaatccgcaaggacgctttcgccg
	Reverse	gctctagatcattgagcctgctgctgtccctgc
HA-KHSRP	Forward	ccggaattcatgtcggactacagcacgggaggac
	Reverse	gctctagatcattgagcctgctgctgtccctgc
Flag- KHSRP	Forward	cggaattcatgtcggactacagcacgggaggac
	Reverse	ccgctcgagtcattgagcctgctgctgtccctgc

2) Primers used for construction of Flag-SUMO1-KSRP aa 68-712

SUMO1-KHSRP	Primer	sequences (5’-3’)
First cycle	Forward	acgcgtcgacggaatccgcaaggacgctttcgccg
	Reverse	ccgctcgagtcattgagcctgctgctgtccctgc
Second cycle	Forward	cccaagctttctgaccaggaggcaaaaccttc
	Reverse	acgcgtcgacccctgtttgttcctgataaacttc

3) Primers for point mutation and NLS deletion mutation of KSRP

h KSRP	Primer	sequence (5’-3’)
KHSRP K87R	Forward	agaattggaggcgatgctgccacga
	Reverse	ggctgcaatctggcgggcccgctgc
KHSRP K359R	Forward	aggcaagatgacgggacagggcccg
	Reverse	gaactgtatccgcacgccagcatca
KHSRP K628R	Forward	aggatcggccagcagccccagcagc
	Reverse	tttgtaatactcttcccaggcctta
KHSRP K244R	Forward	agggccggcctggtcattggcaagg
	Reverse	gcccgcggggatcatgatctcctgc
KHSRP K251R	Forward	aggggcggggagaccattaagcagc
	Reverse	gccaatgaccaggccggccttgccc
KHSRP K435R	Forward	aggtgtgggctggtcatcggccgag
	Reverse	gtgagtggggatggagaaggtcatc
KHSRP K473R	Forward	aggttgttcatcatccggggttcac
	Reverse	gaagttggggtccccgttgggtggc
KHSRP K494R	Forward	aggatcgagggtcctctctgcccag
	Reverse	ttcctcgataagctgcttggcgtgg
KHSRP NLS Del	Forward	ctggcttcccagggagactcaatcagttc
	Reverse	ttggcccccaaaaccaaaatcaggagtgctg
KHSRP S193A	Forward	gccttgacaggagccccagaatctg
	Reverse	cacactgcgctcgggtaggccaccgc
KHSRP S193D	Forward	gacttgacaggagccccagaatctg
	Reverse	cacactgcgctcgggtaggccaccgc

3) Primers used for constructions of lentiviral KHSRP expression, shRNAs for KHSRP, shRNA for

SENP1

	Primer	Sequence (5’-3’)
lentiviral-HA-KSRP	Forward	gctctagagccaccatgtatccttacgacgttccagactatgcaatgtcggactacagcacggg
	Reverse	cggaattctcattgagcctgctgctgtccctgc
KHSRP -shRNA	Forward	ccggtctgaggataaagcaattcattctcgagaatgaattgctttatcctcagtttttg
	Reverse	aattcaaaaactgaggataaagcaattcattctcgagaatgaattgctttatcctcaga
SENP1-shRNA -1	Forward	ccggtagaatactcttgcaataccctcgagggtattgcaagagtattctttttg
	Reverse	aattcaaaaagaatactcttgcaataccctcgagggtattgcaagagtattcta
Ubc9-shRNA	Forward	ccggtgcctacacgatttactgccaactcgagttggcagtaaatcgtgtaggctttttg
	Reverse	aattcaaaaagcctacacgatttactgccaactcgagttggcagtaaatcgtgtaggca

4) Primers for qRT-PCR

miRNAs	Primer	sequence (5’-3’)
GAPDH	Forward	atgaggtccaccaccctgtt
	Reverse	ctcaagggcatcctgggcta
U6	Forward	cgcttcggcagcacatatac
	Reverse	aggggccatgctaatcttct
miR-98-5p	RT-primer	gtcgtatccagtgcagggtccgaggtattcgcactggatacgacaacaat
	Forward	gcctgtgaggtagtaagttgta
let-7i-5p	RT-primer	gtcgtatccagtgcagggtccgaggtattcgcactggatacgacaacagc
	Forward	gcctgtgaggtagtagtttgtg
miR-183-5p	RT-primer	gtcgtatccagtgcagggtccgaggtattcgcactggatacgacagtgaa
	Forward	gcctgtatggcactggtagaat
miR-182-5p	RT-primer	gtcgtatccagtgcagggtccgaggtattcgcactggatacgacagtgtg
	Forward	gcctgtttggcaatggtagaactc
pri-let-7a-1	Forward	tttcaccattcaccctggat
	Reverse	ccaggccataaacaaatgct
pri-let-7a-3	Forward	ttggtggtttcctgcagg
	Reverse	aactgagacccagagaga
RT-let-7a	Forward	gtcgtatccagtgcagggtccgaggtattcgcactggatacgacaactat
RT-U6	Reverse	aggggccatgctaatcttct
qRT-let-7a	Forward	gcctgtgaggtagtaggttg
qRT-Rev primer	Reverse	gtgcagggtccgaggt
